# A tale of two seasons: The link between seasonal migration and climatic niches in passerine birds

**DOI:** 10.1002/ece3.6729

**Published:** 2020-10-20

**Authors:** Alison Eyres, Katrin Böhning‐Gaese, C. David L. Orme, Carsten Rahbek, Susanne A. Fritz

**Affiliations:** ^1^ Department of Biological Sciences Goethe University Frankfurt Germany; ^2^ Senckenberg Biodiversity and Climate Research Centre Senckenberg Gesellschaft für Naturforschung Frankfurt Germany; ^3^ Department of Life Sciences Imperial College London London, Ascot UK; ^4^ Center for Macroecology, Evolution and Climate, GLOBE Institute University of Copenhagen Copenhagen Denmark

**Keywords:** comparative analysis, macroecology, nonbreeding, Passeriformes, seasonal migration, tropics

## Abstract

The question of whether migratory birds track a specific climatic niche by seasonal movements has important implications for understanding the evolution of migration, the factors affecting species' distributions, and the responses of migrants to climate change. Despite much research, previous studies of bird migration have produced mixed results. However, whether migrants track climate is only one half of the question, the other being why residents remain in the same geographic range year‐round. We provide a literature overview and test the hypothesis of seasonal niche tracking by evaluating seasonal climatic niche overlap across 437 migratory and resident species from eight clades of passerine birds. Seasonal climatic niches were based on a new global dataset of breeding and nonbreeding ranges. Overlap between climatic niches was quantified using ordination methods. We compared niche overlap of migratory species to two null expectations, (a) a scenario in which they do not migrate and (b) in comparison with the overlap experienced by closely related resident species, while controlling for breeding location and range size. Partly in accordance with the hypothesis of niche tracking, we found that the overlap of breeding versus nonbreeding climatic conditions in migratory species was greater than the overlap they would experience if they did not migrate. However, this was only true for migrants breeding outside the tropics and only relative to the overlap species would experience if they stayed in the breeding range year‐round. In contrast to the hypothesis of niche tracking, migratory species experienced lower seasonal climatic niche overlap than resident species, with significant differences between tropical and nontropical species. Our study suggests that in seasonal nontropical environments migration away from the breeding range may serve to avoid seasonally harsh climate; however, different factors may drive seasonal movements in the climatically more stable tropical regions.

## Graphical abstract

By examining seasonal niche overlap in migratory and resident bird species, we show that niche tracking varies depending on (a) the perspective in which the question was examined (i.e., from the perspective of the individual migrant or in comparison with resident species, (b) the direction of migration (i.e., whether the species is moving from the breeding or non‐breeding range), and (c) geographic location. Altogether, these results suggest that the drivers of migration vary depending on direction of migration as well as geographically, offering explanations for the mixed results from previous studies.

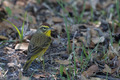

## 1 INTRODUCTION

The worldwide spectacle of animal migration has fascinated people for thousands of years (Thompson, 1907). However, despite the considerable attention that has been given to migration (Greenberg & Marra, 2005), it remains unclear what drives these seasonal movements. One hypothesis proposed to explain the seasonal movements of migratory species is that they track preferred climatic conditions to avoid harsh seasonal climates (Joseph & Stockwell, 2000). Understanding the extent to which migratory species track climatic conditions throughout the year has important implications for understanding the evolution of migration (Nakazawa & Peterson, 2004; Winger, Auteri, Pegan, & Weeks, 2019); the factors affecting species' distribution (Boucher‐Lalonde, Kerr, & Currie, 2013); and the responses of species to past or future climate change (Thomas et al., 2004). These questions are particularly relevant for birds as ~20% of all species are migratory, changing distribution throughout the year (Eyres, Böhning‐Gaese, & Fritz, 2017; Kirby et al., 2008). In this study, we investigate the relationship between migratory behavior and the climatic conditions occupied by different species in each season using a phylogenetic comparative framework.

Climatic conditions are dynamic with one notable pattern of climatic variation being seasonal variations, which are most pronounced in temperate regions. Migratory species might be expected to move to track climatic conditions directly if they move to avoid harsh climatic conditions that they cannot physiologically tolerate (Joseph & Stockwell, 2000; Somveille, Rodrigues, & Manica, 2015). Although there is evidence that birds are able to acclimatize to tolerate extremely low or high temperatures through increasing metabolic rates and behavioral adaptations, respectively, these are energetically expensive and require significant increases in energy and water intake (Hart, 1962; Riddell, Iknayan, Wolf, Sinervo, & Beissinger, 2019). Therefore, there may be limits to the climatic conditions under which a species is able to survive (Canterbury, 2002; Khaliq, Hof, Prinzinger, Böhning‐Gaese, & Pfenninger, 2014). As well as direct physiological limitations, migrants might track climatic conditions in order to pursue seasonally available resources (Greenberg & Marra, 2005; Luis Tellería, Ramirez, & Pérez‐Tris, 2008; Thorup et al., 2017). Alternatively, migratory species may occupy different climatic conditions in each season if they move to avoid extreme climatic conditions rather than to track specific conditions (Newton, 2008), have different seasonal requirements (Spencer, 1982), or because movement is driven by factors other than climate, for example, nest predation (McKinnon et al., 2010), reduced parasite load (Piersma, 1997) or energy availability and competition for limited resources (Somveille, Rodrigues, & Manica, 2018). To assess whether seasonal migrants track the climatic conditions in their breeding grounds when moving to nonbreeding grounds and vice versa, studies have increasingly used the climatic niche concept (Boucher‐Lalonde et al., 2013; Laube, Graham, & Böhning‐Gaese, 2015). This describes the climatic conditions within which a species can maintain a viable population (Bonetti & Wiens, 2014; Pearman, Guisan, Broennimann, & Randin, 2008).

Mixed support has been found for climatic niche tracking (see Engler et al. (2017) and Table 1 for reviews of the topic). For example, although Joseph and Stockwell (2000) found that the Swainson's flycatcher tracks its niche throughout the year, subsequent studies have shown that this is not the case for all migratory species (Laube et al., 2015; Martinez‐Meyer, Townsend Peterson, & Navarro–Sigüenza, 2004; Nakazawa & Peterson, 2004; Ponti, Arcones, Ferrer, & Vieites, 2020; Zurell, Gallien, Graham, & Zimmermann, 2018). Migratory species in the family Parulidae (American wood‐warblers) were found to track their niche to a greater extent than resident species (Gómez, Tenorio, Montoya, & Cadena, 2016), whereas a global study found little evidence for seasonal temperature tracking of migratory compared to resident species (Dufour et al., 2020). Most other studies have focussed on migratory species only, showing much regional and species‐level idiosyncrasy in seasonal niche overlap (Zurell et al., 2018) and a trade‐off between tracking temperature across seasons, access to seasonal resources, and the cost of longer migratory distances (Somveille, Manica, & Rodrigues, 2018). However, as previous studies were carried out on different groups of birds, in different geographic regions, and using a variety of different methods (Table 1), generalization is difficult and the reasons behind the observed variation in niche tracking across species remain unclear.

**Table 1:** Review of the major studies that have tested for seasonal niche tracking in birds

Reference	Study species	Region	Niche parameters	Covariates	Comparison	Main finding
Dufour et al. (2020)	9,819 species (migratory and resident)	Global	Temperature		Non‐migration of migratory species; resident species	Niche switching
Ponti et al. (2020)	355 migratory species	Eurasian‐African flyways	Temperature, precipitation	Migratory distance	Non‐migration of migratory species	Niche switching
Zurell et al. (2018)	717 migratory species	Holarctic	Temperature, precipitation, NDVI, Land cover	Ecological traits (body mass and diet) Range size Range position Region	Similarity tests (comparison to migrating at random)	Niche tracking in 65%–95% of species^a^
Gómez et al. (2016)	54 resident and 49 migrant species of wood‐warblers (Parulidae)	New World	Temperature, precipitation, topography		Resident species	Niche tracking
Pérez‐Moreno, Martínez‐Meyer, Soberón Mainero, Rojas‐Soto (2016)	13 migratory species	New World	Temperature and precipitation		Non‐migration from nonbreeding range	Niche tracking and switching
Laube et al. (2015)	26 species of migratory and resident *Sylvia* warblers	Europe, Africa and western Asia	Temperature, precipitation, NDVI, land cover classification	Migratory distance	Non‐migration of migratory species Resident species	Niche switching
Martinez‐Meyer et al. (2004)	9 species of migratory and resident *Passerina* buntings	North America and northern South America	12 climatic parameters: temperature, precipitation, radiation etc.		Interpredictability of seasonal niches	Clear niche tracking in 40% of migrants
Nakazawa and Peterson (2004)	21 migratory species	New World	Temperature Frost days Vapor pressure		Interpredictability of seasonal niches	Clear niche tracking in 47%, clear switching in 4%
Joseph and Stockwell (2000)	*Myiarchus swainsoni* (Austral migrant)	South America	Temperature		Interpredictability of seasonal niches	Niche tracking

Note

Details on study system and method are given with the main findings for each study. In particular, we indicate how the experienced seasonal climatic niche similarity of migratory species was assessed, that is, by comparisons with hypothetical scenarios (no‐ migration of migratory species or random migration), comparison with resident species, or by assessing whether a niche model trained on one season could predict the niche in the other season (interpredictability).

^a^ Less evidence of niche tracking when only climatic variables considered.

In addition to this evidence for variation in the degree of climatic niche tracking, there is some indication that niche tracking could vary depending on direction of migration, that is, whether birds are moving toward their breeding or nonbreeding range (Martinez‐Meyer et al., 2004; Nakazawa & Peterson, 2004). Somveille et al. (2015) showed that species richness in birds is influenced by different climatic factors in the breeding and nonbreeding season, suggesting that birds might leave breeding areas to avoid climatic seasonality there, whereas leaving the nonbreeding range may be associated with exploiting seasonal resource availability in breeding areas. This asymmetry has additionally been predicted under several hypotheses for the evolution of migration, for example, theories that propose that migration has evolved to avoid seasonal habitats in the breeding season (Salewski & Bruderer, 2007; Winger et al., 2019). Despite asymmetry in niche tracking being expected from theory, this is yet to shown explicitly.

Most previous studies have tested the ability of migratory birds, in particular long‐distance migrants (Boucher‐Lalonde et al., 2013; Somveille et al., 2015; Zurell et al., 2018), to track a niche across seasons by comparing to a null expectation (Table 1). A variety of null expectations have been used, for example, by comparing whether the niche overlap is greater than if species did not migrate but stayed in each of their seasonal ranges (Laube et al., 2015), if species migrated to a random location (Zurell et al., 2018) or if species migrated to seasonal ranges derived from a simulation model controlling for the migration options available to each species (Somveille, Manica, et al., 2018). Although these comparisons provide important information about niche tracking from the perspective of each migratory species, they do not determine why some species migrate and others do not. In contrast to migrants, resident species stay in one location and tolerate the entire annual range of climatic conditions in their breeding regions (Hafthorn, 1989). Seasonal migration has evolved multiple times in birds as a whole, and many genera and families actually include closely related migratory and resident lineages, indicating frequent evolutionary transitions between migratory and resident behavior (Phillips, Töpfer, Böhning‐Gaese, & Fritz, 2018; Winger, Barker, & Ree, 2014). Therefore, whether species migrate to track seasonal climate is only one side of the question, with the other being to what degree resident species do not track seasonal climate.

A comparison of the occurrence–climate relationships among migratory and closely related resident species in a phylogenetic comparative framework therefore adds an important additional perspective of shared biogeographic history. This perspective has been largely absent from the literature so far (Table 1; but see Gómez et al. (2016) and Dufour et al. (2020)). In this study, we explicitly test seasonal niche tracking both within migratory birds and across migratory and resident species. We do so using a large dataset comprising 437 extant species in eight passerine clades found across the world (Figure S1) and controlling for biogeographic range size and phylogenetic effects. In addition, we use a consistent new classification of migratory behavior (Eyres et al., 2017), which is based on descriptions of migratory behavior that can identify partially migratory behavior even when range maps, which have previously been used to identify migrants, do not show seasonal variation; our analysis also includes a greater diversity of movement types (i.e., both short‐ and long‐distance migrants) than most previous studies (Ponti et al., 2020; Somveille, Manica, et al., 2018; Somveille et al., 2015; Zurell et al., 2018). Further, we quantify seasonal niche overlap from geographic occurrences using a new database containing up‐to‐date maps of species’ breeding and nonbreeding distributions. This database was specifically created to produce the best possible range map for each species classified as at least partially nonresident in Eyres et al. (2017), that is, with a focus on migratory or nomadic species.

We test for climatic niche tracking by comparing the observed climatic overlap of migratory species with two types of null expectations. For the first test, we compare observed niche overlap of migrants with the overlap they would experience if they did not migrate and stayed in either the breeding or the nonbreeding range year‐round. Note that this tests each migratory direction separately, allowing us to test whether there are different drivers for leaving the breeding or nonbreeding range (as discussed above). For our second method, we compare the observed overlap of migratory species with the overlap of closely related resident species.

These tests are carried out within a phylogenetic comparative framework, accounting for two biogeographic factors that have not been consistently considered in previous studies (Table 1). First, the degree to which species track climatic conditions is expected to vary with breeding location because climate seasonality increases with latitude (Archibald, Bossert, Greenwood, & Farrell, 2010). The combined analysis of tropical and nontropical breeding species may therefore obscure any signal of climatic niche tracking (Zurell et al., 2018), so we control for the effects of breeding location (within versus outside the tropics). Second, most previous studies have not taken geographic range size into account in analyses of niche tracking. Within long‐distance migrants, range size has been shown to be significantly positively related to seasonal niche overlap (Zurell et al., 2018). Therefore, we control for range size in our analyses.

**Figure 1:**
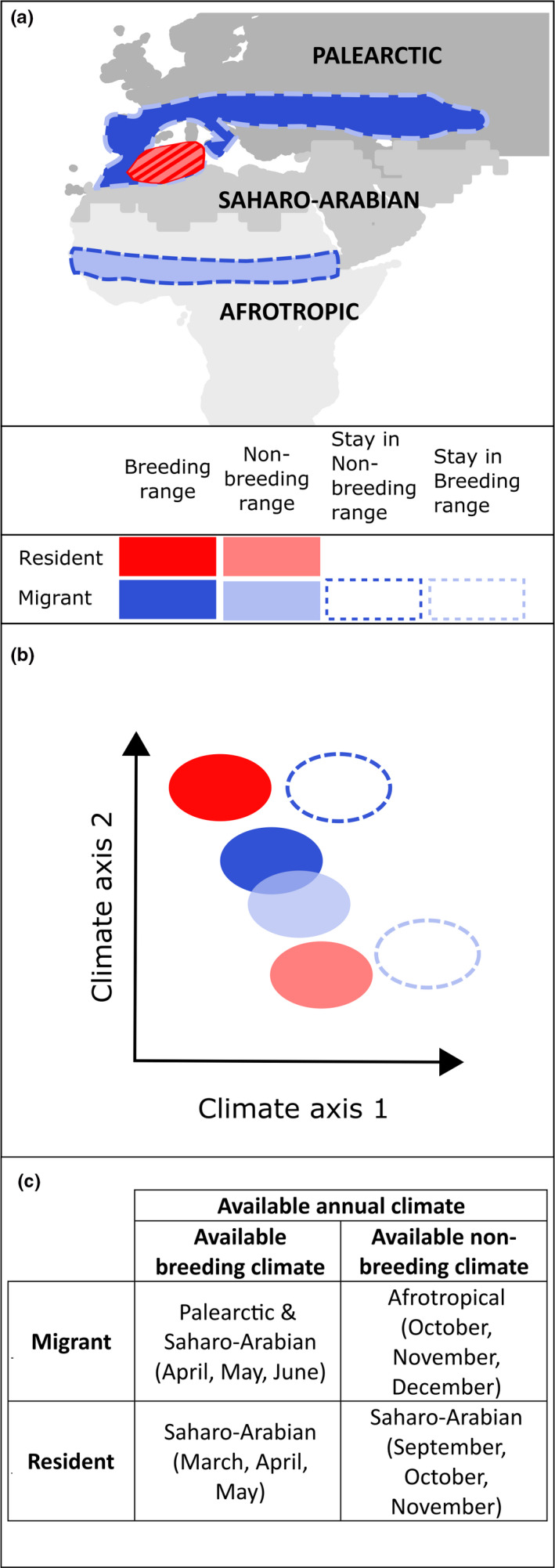
Schematic showing examples of a nontropical migratory and a nontropical resident species: geographic distributions in breeding and nonbreeding season (a), expectations of niche overlap in climatic space under the hypothesis of niche tracking (b) and zoogeographic realms and months we used to define the climatic space available to each example species in each season (c). Expectation 1 (distributions and niches shaded in blue versus those surrounded by dashed blue lines): If migrants track climatic conditions, it is expected that the seasonal niche overlap is greater than if they did not migrate and stayed in either the breeding or nonbreeding range year‐round. Expectation 2 (distributions and niches shaded in blue versus those shaded in red): If migrants track climatic conditions, it is expected that the breeding and nonbreeding niches are more similar in climatic space (higher overlap) than those of residents. Hatched regions in panel a depict overlapping breeding and nonbreeding areas of an example resident species

The hypothesis that migratory species move to track seasonal climatic niches (Figure 1) generates the following predictions, each of which we test against appropriate null expectations:

1. If migrants track seasonal climatic niches, we expect the overlap between seasonal climatic niches (i.e., breeding versus. nonbreeding) experienced by migrants to be greater than the hypothetical seasonal niche overlap that would arise if a migratory species did not migrate (i.e., stayed in the breeding or nonbreeding range year‐round; blue species in Figure 1a,b), when controlling for range size and phylogeny (Laube et al., 2015). In addition, we expect an effect of breeding location: The previous expectation should hold more strongly for species breeding outside the tropics, but the observed and hypothetical seasonal niche overlap might not differ for species breeding in the tropics where climatic conditions remain relatively stable year‐round.
2. If migrants track seasonal climatic niches, we expect higher overlap between breeding versus nonbreeding climatic niches for migratory species than for resident species (contrast blue and red species in Figure 1a,b), when accounting for range size and phylogeny (Gómez et al., 2016). In addition, we expect an interaction between breeding location and migratory behavior: Migrants might have larger seasonal overlap than residents only if breeding in nontropical regions due to the stronger climatic seasonality there. No difference in seasonal niche overlap is expected between migrants and residents breeding in the tropics if climatic conditions remain relatively stable year‐round there.

## 2 METHODS

We selected eight monophyletic clades from across the Passeriformes that contained a mixture of migratory and resident species and were distributed globally (Table 2 and Figure S1). Each clade was selected to have similar orders of species richness (approximately 50–80 species each), at least 30% nonresident species, and a well‐resolved molecular phylogeny. Species names followed IOC taxonomy V 3.1 (Gill & Donsker, 2012). Classification of migratory behavior followed Eyres et al. (2017). This classification scheme is based on descriptions of movements from the handbook of the birds of the world (del Hoyo, Elliott, Sargatal, Christie & de Juana, 2019) and was chosen over classifying migratory behavior directly from range maps because migratory behavior is not always evident in range maps, for example, in partially migratory species. Previous studies have focused on migratory species with spatially distinct seasonal breeding and nonbreeding areas, and might therefore be biased in terms of expected niche overlap.

**Table 2:** Details of study clades

Clade	Genera	Passeriform lineage	Total Species	Number of species by migratory type					Number of included Tropical breeders		Number of included Nontropical breeders	
				Resident	Directional migrant	Dispersive migrant	Nomadic	Unknown	Resident	Directional migrant	Resident	Directional migrants
Xolmiini Tyrant flycatchers	*Muscisaxicola, Knipolegus, Xolmis, Agriornis, Myiotheretes, Lessonia, Cnemarchus, Heteroxolmis, Hymenops, Neoxolmis, Polioxolmis, Satrapa,*	Suboscines: Tyranni, Tyrannidae	48	28	20	0	0	0	24	15	3	5
*Vireonidae* Vireos, Greenlets, and Allies	*Vireo, Hylophilus, Vireolanius, Cyclarhis*	Oscines: Corvoidea	53	40	13	0	0	0	13	3	18	10
*Corvidae* Crows and Ravens	*Corvus, Coloeus*	Oscines: Corvoidea	47	34	9	3	1	0	13	0	16	9
*Hirundinidae* Swallows and Martins	*Hirundo, Petrochelidon, Cecropis, Progne, Tachycineta, Riparia, Psalidoprocne, Notiochelidon,* *Ptyonoprogne, Delichon, Atticora, Phedina, Pseudochelidon, Stelgidopteryx, Alopochelidon, Cheramoeca, Haplochelidon, Neochelidon, Pseudhirundo*.	Oscines: Sylvioidea	88	32	43	12	0	1	17	28	14	11
*Turdus* Thrushes	*Turdus, Nesocichla, Psophocichla*	Oscines: Muscicapoidea, Turdidae	81	53	25	3	0	0	25	4	19	21
*Oenanthe* Wheatears, Chats, and Allies	*Oenanthe, Saxicola,* *Monticola, Myrmecocichla, Emarginata, Pentholaea, Thamnolaea, Campicoloides, Pinarochroa*	Oscines: Muscicapoidea, Muscicapidae	70	44	23	2	0	1	15	0	25	20
*Setophaga* Wood‐warblers	*Setophaga, Myiothlypis, Myioborus, Basileuterus, Cardellina, Catharopeza*	Oscines: Passeroidea, Parulidae	80	50	30	0	0	0	19	0	15	27
*Cardinalidae* Cardinals, Grosbeaks, and Allies	*Piranga, Passerina, Pheucticus, Habia,Chlorothraupis,* *Amaurospiza, Cardinalis, Cyanocompsa, Granatellus* *Caryothraustes, Cyanoloxia* *Periporphyrus, Rhodothraupis, Spiza*.	Oscines: Passeroidea	51	35	15	1	0	0	13	3	20	12

Note

Clades are in taxonomic order following (IOC World Bird List (v 3.1) (Gill & Donsker, 2012). Genera are listed by species number from highest to lowest. Migratory classification follows Eyres et al. (2017). Number of total species includes two extinct species that were not scored for migratory behavior. For the residents and directional migrants only, we also indicate how many species are tropical breeders or nontropical breeders. Tropical breeders are those which have at least 10% of their breeding range in tropics. Note that some species had to be excluded from niche calculations because of extremely small range sizes, so tropical and nontropical species numbers do not add up to the clade total.

For each species, we characterized the breeding and nonbreeding climatic niches using seasonal distribution and climate data. Breeding time is species‐specific, so we determined the three peak breeding months for each species individually using information from the literature (del Hoyo et al., 2019, and others; see Table S1 for details). Where no information was available on the breeding months, these were chosen using information from congeneric species breeding in the same geographic region (31 of 437 species in the final analyses; for details, see Table S1). The three nonbreeding months for each species were defined as 6 months later than the breeding season, a somewhat arbitrary decision given the different degree of climatic seasonality and migratory timing in different regions and species, but chosen to be globally consistent across all species.

### 2.1 Range maps and climatic datasets

To characterize climatic niches, geographic distributions for the breeding season were obtained from the *Copenhagen global avian distributional database* (Holt et al., 2013, updated version from June 2014). This is an extensive database mapping a conservative extent of occurrence during the breeding season at a 1° latitudinal–longitudinal resolution for each species based on museum specimens, published sight records, and the spatial distribution of habitats between documented records, which have been validated by ornithological experts. It is therefore superior to existing polygon range maps because it gives a more precise estimate of species' occurrences. Since no dataset like this exists that is consistent for all species in the nonbreeding season, we compiled a new set of nonbreeding distributions of migratory species as extent‐of‐occurrence polygons, the *GeoMiB database (Geographic distributions of migratory birds* v. 1.1) and sampled this to the same resolution as the breeding ranges. This dataset was created specifically to produce the best possible range map for each species classified as at least partially nonresident (migratory or nomadic) in Eyres et al. (2017) by combining the best source for seasonal range maps for each region into a global seasonal range map for each species (see Table S2 and supplementary information for more details). Final species occurrences were therefore seasonal presences in 1° latitude–longitude grid cells where species were recorded in the Copenhagen database (breeding and year‐round, with the difference among the two determined from the GeoMiB range maps) or where >5% of the grid cell area was covered by species' range maps from the GeoMiB database (nonbreeding). Any regions mapped as year‐round were assigned both the breeding and the nonbreeding months for each species (note that such areas do not only occur in resident species but are also common in many partial migrants).

As the two datasets were compiled using slightly different methodology, all combined range maps were manually checked and obvious deviations from other range map compilations (BirdLife International & NatureServe., 2016; del Hoyo et al., 2019) were either removed as errors or verified as an improvement. Our procedure ensured that the combined dataset represents the best estimate of each species' occurrence in breeding and nonbreeding seasons, but it still reflects generally lower spatial detail for the nonbreeding areas, highlighting a lack of knowledge of species’ nonbreeding areas compared to their breeding areas that might affect seasonal niche overlap comparisons. Finally, although any such extent‐of‐occurrence data are not ideal for quantifying climatic niches (Graham & Hijmans, 2006), they represent the most consistent and accurate coverage of species’ ranges that are currently available at a global scale (particularly in the tropics) and across a large number of species (Meyer, Kreft, Guralnick, & Jetz, 2015).

Monthly climate data for all zoogeographic realms (Holt et al., 2013) inhabited by the study species (Figure S1) were obtained from the *CliMond raw climate data* dataset (averages from 1961 to 1990, 10′ resolution) (Kriticos et al., 2012) and spatially averaged into the same grid as the occurrence data. For each species, the extracted climatic data for each occupied grid cell in the range gave us three monthly values for each variable in either season. The following climatic variables for each month were used: minimum and maximum of daily temperatures averaged within each month, total monthly precipitation, mean daily humidity of each month, and mean daily relative humidity at 9 a.m. and at 3 p.m. for each month. These six climatic variables were chosen as ecologically relevant descriptors of global climate including extremes of temperature and water availability (Petitpierre, Broennimann, Kueffer, Daehler, & Guisan, 2017). Minimum and maximum values of temperature were chosen rather than mean values as the climatic conditions tolerated by species are often not normally distributed (Evans, Smith, Flynn, & Donoghue, 2009). Including the minimum and maximum is more likely to capture the full range of conditions that a species can tolerate (Budic & Dormann, 2015).

### 2.2 Niche metrics and explanatory variables

To test prediction one (Figure 1, blue species), we quantified the climatic niche overlap of migratory species between seasons from the seasonal occurrence data and compared it to two hypothetical situations or null expectations: the overlap that would result if a species stayed in the breeding range for the whole year, the overlap that would result if a species stayed in the nonbreeding range for the whole year. To test prediction two, we calculated and compared the overlap in climatic niche between seasons for resident species with that of migratory species (Figure 1, red species versus blue species, respectively). All analyses were carried out in R version 3.6.1 (R Core Team, 2014).

We characterized seasonal niche overlap as a measure of niche similarity that compares the entire climatic niche space experienced by the species in each season. Following Broennimann et al. (2012), principal component analysis (PCA) was used to incorporate information and identify the major axes of variation from all six climatic variables, reduce variable redundancy, and finally create a two‐dimensional climatic space in which niche overlap could be measured. As different climatic factors might be important for determining each clade's distribution, we carried out a PCA for each clade individually (see Tables S3 and S4). Through inclusion of not only the species' occurrences but also the climate available to the clade in each clade‐wide PCA, the method to calculate niche overlap accounts for different availability of climatic conditions through time and among species through calculation of “climatic occupancy values” (Broennimann; for details, see Supplementary materials, Methods). This makes it particularly appropriate for testing for niche similarity across many species at different time points (Zurell et al., 2018) or in different geographic locations (Petitpierre et al., 2012). The climate available to a species in a season was defined as the climate across all zoogeographic realms that the species inhabits in that season; the climate available to the clade as a whole was defined as all the zoogeographic realms that any member of the clade inhabits (see Figure 1c for an example species) (Holt et al., 2013, details in Supplementary methods).

Each clade‐wide PCA comprised the climatic conditions experienced by and available to all members of that clade in both the breeding and the nonbreeding season. The overlap between breeding and nonbreeding niches was then calculated for each species based on the climatic occupancy values using Schoener's D, a measure that varies between 0 (no overlap) and 1 (complete overlap) (Warren, Glor, & Turelli, 2008). Alternative approaches exist which allow quantification of climatic niches in more dimensions, for example, the hypervolume approach (Blonder, Lamanna, Violle, & Enquist, 2014). However, for each of our clade‐wide PCAs the first two components explained >86% of variation in the data (Table S3) and exhibited high factor loadings for the two most divergent niche aspects (temperature/radiation versus. precipitation/humidity, Table S4). Therefore, approaches considering more niche dimensions based on the same climatic variables would not add significant insight into overlap.

In order to test whether the breeding location affects niche overlap between seasons, species were categorized as tropical breeding if at least 10% of the breeding range occurred between 23.5°N and 23.5°S; else, as nontropical breeding. Division into these two categories was chosen rather than using a continuous latitude variable because we expect any potential effect of breeding location to reflect an underlying effect of regional climatic seasonality; the tropical–temperate split represents the most striking difference in climatic seasonality globally, whereas latitude is related to climatic seasonality differently in the north versus south hemispheres (Archibald et al., 2010). Although the threshold of 10% is arbitrary and our definition of tropical breeder is generous, this ensured that all species classified as nontropical breeders really experienced nontropical climatic seasonality.

Geographic range size was determined for each species as the sum of the total land area within all grid squares occupied by the species in the breeding distribution and all the grid squares occupied by the species in the nonbreeding distribution (i.e., year‐round distributions were counted twice, because year‐round occurrences also enter the niche calculations twice, once for the breeding and once for the nonbreeding months). Range size was log‐transformed in all analyses because the data were not normally distributed (as determined by the Kolmogorov–Smirnov test, *p* < .001).

### 2.3 Comparative analyses across species

In total, our selected clades contained 518 extant species displaying a variety of migratory behaviors: dispersive migration (*n* = 21), directional migration (*n* = 178), nomadism (*n* = 1), residency (*n* = 316), and species with unknown movement behavior (*n* = 2) (Table 1). We omitted dispersive migrants, defined as those where individuals make regular postbreeding movements in any geographic direction from breeding sites (Newton, 2008), nomadic species (which perform nonseasonal movements), and those with unknown movement behavior from our analyses, because it is unlikely that seasonal range maps are able to accurately represent distribution patterns of these species (24 species in total). Five additional species were omitted because they lacked distribution data, while 51 species were additionally omitted from analyses because they had a too small range size to calculate niche metrics using our methods (see Supplementary Materials, Table S1 for full species list). Final analyses were carried out on 437 species. Eight species included in the analysis were defined as directional migrants but only had year‐round distribution data available.

To determine whether geographic range size influenced seasonal niche overlap, we tested whether range size differed between categories of movement behavior and for a relationship between range size and seasonal niche overlap using linear mixed‐effects models. These analyses showed significant relationships (Figure S2, details in Supplementary material, Methods), so geographic range size was included in all subsequent models.

To test prediction one (i.e., that migratory species increase seasonal overlap by migrating away from their breeding or nonbreeding range), we used paired Wilcoxon's tests to compare the overlap between observed seasonal niches with two measures of hypothetical overlap, assuming the species stayed in one of the two seasonal ranges (Laube et al., 2015). To determine whether the effect of migration was influenced by breeding location, this analysis was carried out separately on tropical and nontropical breeding species. *p*‐Values were corrected with Bonferroni's correction to account for multiple testing within each breeding location. To check that results were not unduly influenced by differences in range size, we additionally constructed two linear mixed‐effects models in which the response variable was the difference between the observed niche overlap and each hypothetical overlap (i.e., we ran separate models for staying in the breeding range and for staying in the nonbreeding range), and the fixed effect was the difference between the observed range size and the range size that occurred in the respective hypothetical scenarios. As the values for difference in seasonal range size were on a very different scale to other variables, they were first scaled to be between −1 and 1 using the rescale function from the *plotrix* package (Lemon, 2006). To control for phylogeny, clade was included as a random effect.

To test prediction two (i.e., that migratory species experience higher seasonal niche overlap than closely related resident species), analyses of seasonal niche overlap across migratory and resident species were performed using linear mixed‐effects models. Clade was included as a random effect to control for phylogenetic effects, with random intercepts allowed for each clade. To test whether seasonal niche overlap differed between migratory and resident species, and whether this relationship was geographically consistent, the fixed effects of migratory status (resident or migratory), breeding location (tropical or nontropical), and geographic range size were tested on seasonal niche overlap. Because we expected that the difference in niche tracking between migratory and resident species may vary depending on breeding location, we fitted an interaction between breeding location and migratory status in our analyses. In addition, we fitted a more complex model including all two‐way interactions with range size, as the each of the effects of migratory status and breeding location on niche overlap might be influenced by range size differently. Although the Akaike information criterion (AIC) values show the more complex model is more strongly supported, this support was only marginal (delta AIC = 3.8). As the results from these two models are qualitatively similar, we report the simpler one in the main text and the model with all two‐way interactions in the supplement. For each model, we calculated the marginal and conditional *R* ^2^ values (i.e., the variance explained by the fixed effects only and by the entire model, respectively) as a measure of goodness of fit of the final models (Nakagawa & Schielzeth, 2013).

To check for clade‐specific effects, we additionally fitted linear models in which clade was included as a fixed effect. Firstly, we fitted a model with breeding location, migratory status, and clade plus their two‐way and three‐way interactions, controlling for range size (but in this case opting for the simpler model where no interactions with range size were fitted). As there are not many species in some of these categories for a few clades (see Table 1, Figure 3), we additionally ran a second model without breeding location. For further information, see supplementary material.

To control for phylogenetic relationships within clades more explicitly than the models described above which only control for clade effects, we additionally fitted equivalent models using phylogenetic generalized least‐squares regression analyses (PGLS). PGLS analyses were conducted using the *caper* package in R (Orme etal., 2014) (details in Supplementary material, Methods).

## 3 RESULTS

### 3.1 Prediction 1: Seasonal niche overlap of migratory species

Overall, observed seasonal niche overlap in climate experienced by migratory species was found to vary from *D* = 0 (no overlap) to *D* = 0.78; 83% of the D values were lower than 0.5, indicating generally low niche tracking within migratory species. The hypothetical seasonal overlap that would be experienced by migrants if they stayed in the breeding or nonbreeding ranges year‐round ranged from *D* = 0 to *D* = 0.80 and from *D* = 0 to *D* = 0.83, respectively. For both cases, more than 75% of *D* values were lower than 0.5.

**Figure 2:**
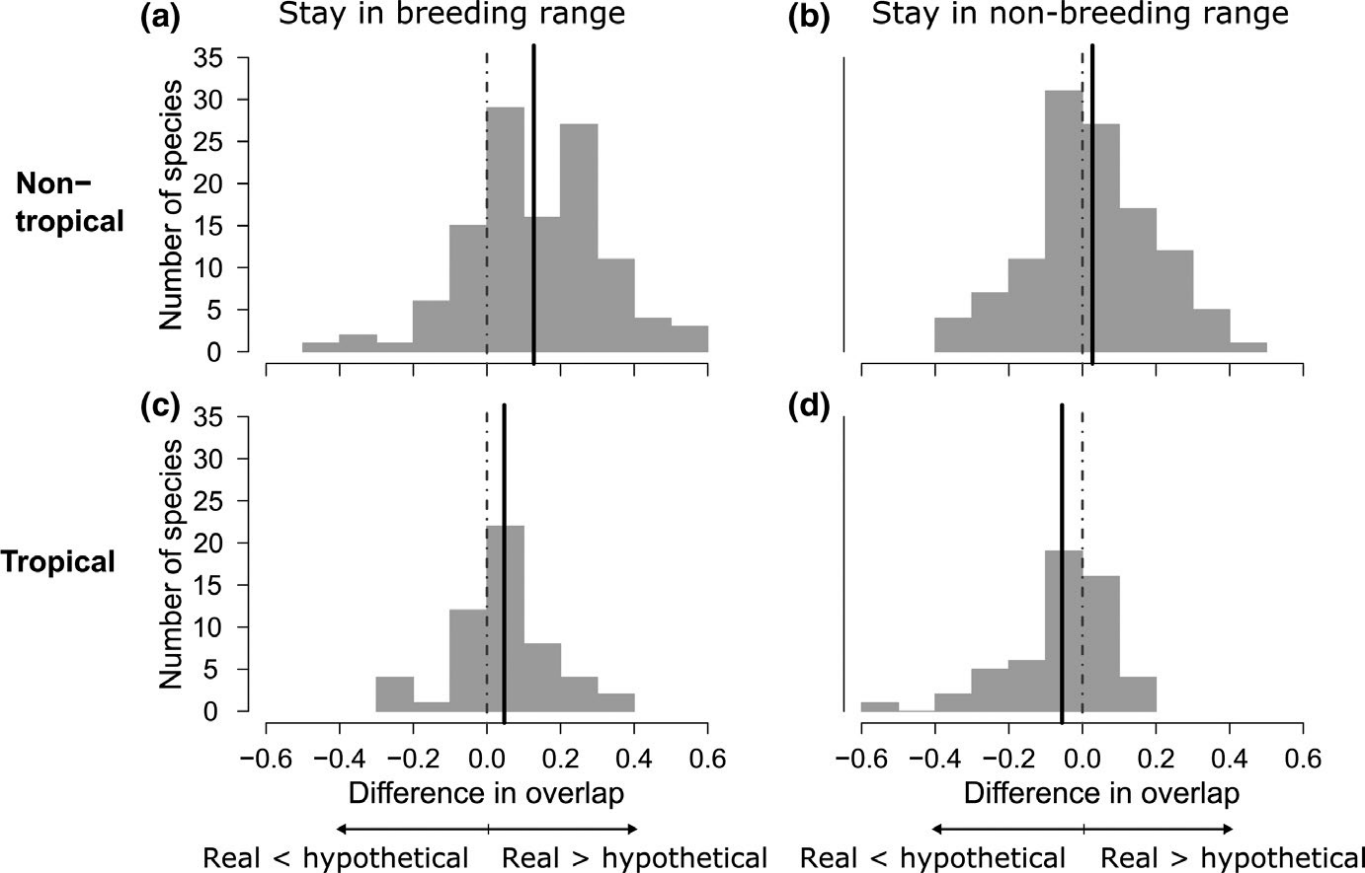
Frequency distributions of differences in niche overlap across migratory species, contrasting the experienced seasonal overlap to hypothetical overlap if migratory species did not migrate but rather stayed year‐round in either the range they occupy in the breeding season (a and c) or nonbreeding season (b and d). This is shown for species breeding outside of the tropics (*N* = 115, a and b) and species that breed at least partly (>10% of breeding range) in the tropics (*N* = 53, c and d). Only directional migrants were considered. We measured observed overlap given migration minus hypothetical overlap assuming no migration. If species track their climatic niche across seasons, positive values are expected: Dotted gray line shows 0 (no difference), and solid black line indicates mean observed difference for each scenario

Contrary to prediction one (Figure 1), the observed seasonal niche overlap in migratory species was not consistently greater than the hypothetical overlap if migrants were to stay in either the breeding or nonbreeding range year‐round, and the results did not differ strongly by breeding location (Figure 2). Instead, whether migratory species increased seasonal niche overlap by migrating was found to vary depending on whether they were migrating away from their breeding or nonbreeding location. As expected, under climatic niche tracking we found that observed overlap was significantly larger than hypothetical overlap if species stayed in the breeding range year‐round, for both nontropical and tropical breeding species (Figure 2a,c, paired Wilcoxon's tests, *p* < .001, *df* = 114; *p* = .006, *df* = 52, respectively). However, this was not the case if species stayed on the nonbreeding range year‐round (nontropical breeding species: Figure 2b, paired Wilcoxon's test, *p* = .142, *df* = 114). In fact, the overlap for tropical breeding species was significantly smaller if they migrated than if they stayed on the nonbreeding range year‐round (Figure 2d, paired Wilcoxon's test, *p* = .022, *df* = 52). We found that the seasonal difference in range size had no significant effect on any of these observed niche overlap patterns in migratory species (Figure S3; linear mixed‐effects models, all *p*‐values > 0.11).

### 3.2 Prediction 2: Comparison of seasonal niche overlap between migratory and resident species

Overall, seasonal overlap values for resident species varied from *D* = 0 to *D* = 0.89 (for comparison, migratory species varied from *D* = 0 to *D* = 0.78). The *D* values for both resident and migratory species were heavily left skewed with 71% and 83% of overlap values being lower than 0.5, respectively.

**Figure 3:**
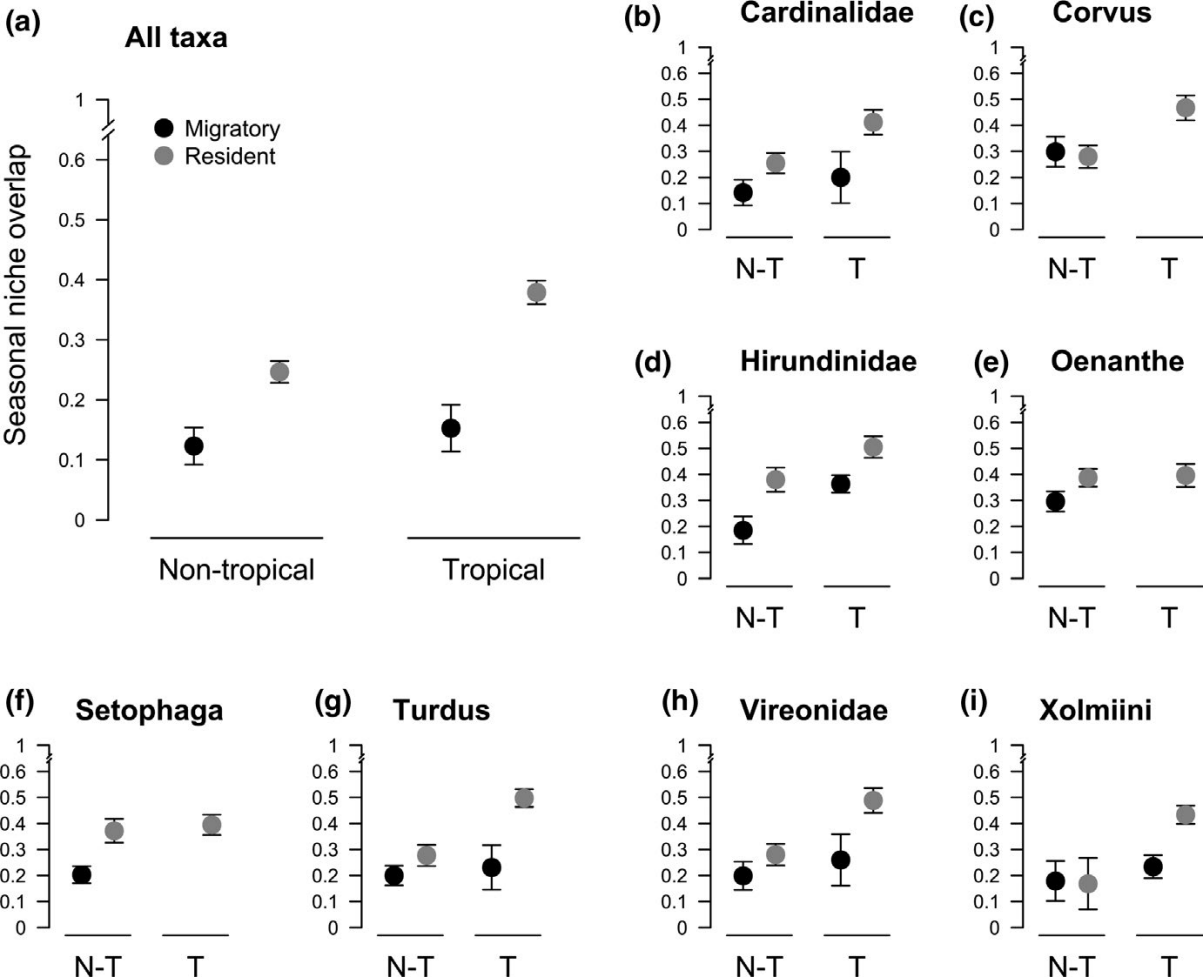
Predicted means and 95% confidence intervals for seasonal niche overlap across migratory (black) and resident (gray) species, separately for nontropical and tropical species (tropical species have at least 10% of breeding range in the tropics). Predictions come from two different models. (1) A linear mixed effector model in which migratory status, breeding location, and their interaction, as well as range size (log‐transformed), were included as fixed effects and clade was included as a random effect (panel a, Table 3). (2) A linear model in which clade (and its two and three‐way interactions with migratory status and breeding location) is included as a fixed effect (panels b‐i show clade‐specific predictions, Table S6). (*N* = 437). Values of seasonal niche overlap can vary from 0 (no overlap) to 1 (complete overlap)

In contrast to our second prediction, migratory species overall had significantly lower niche overlap between the climate experienced during breeding and nonbreeding season than resident species when controlling for clade and range size (Figure 3, Table 3). Tropical species had significantly higher overlap than nontropical species (Figure 3, Table 3). Contrary to expectations there was no significant interaction of breeding location and migratory behavior (Table 3), the (nonsignificant) effect was the opposite to initial expectations as tropical migratory species were found to differ more in seasonal overlap from tropical resident species than nontropical migrants versus nontropical residents (Figure 3). As expected, species with larger range sizes had significantly larger seasonal niche overlap (Table 3). The results of the more complex model including all two‐way interactions were qualitatively similar to the simpler model above; however, breeding location was no longer significant as a main effect. Instead, the interaction between breeding location and migratory status was significant (Table S5). In addition range size interacted significantly with both migratory behavior and breeding location (Table S5; for details, see supplementary materials).

**Table 3:** Model coefficients, *R* ^2^, *p*‐values, and sample sizes of linear mixed‐effects model with seasonal niche overlap as response variable

Predictors	Seasonal niche overlap		
	Estimate (*SE*)	*T* statistic	*p*
(Intercept)	−0.84 (0.10)	−8.41	**<.001**
Migratory status (Resident)	0.11 (0.03)	4.11	**<.001**
Breeding_location (Tropical)	0.08 (0.03)	2.55	**.011**
Log (Range size)	0.07 (0.01)	11.40	**<.001**
Migratory_status (resident)* Breeding_location (Tropical)	0.06 (0.04)	1.51	.131
Random effects			
*σ* ^2^	0.03		
τ_00 Clade_	0.00		
ICC	0.03		
*N*_Clade_	8		
Observations	437		
Marginal *R* ^2^/Conditional *R* ^2^	0.361/0.379		

Note

Predictor variables included fixed effects for migratory status (resident coefficient values shown here), breeding location (tropical breeding coefficient values shown here), range size (logged), and the interaction between migratory status and breeding location (indicated by *). The model also included clade as a random effect. *N* = 437. Bold values indicate significance at *p* < .05.

Our results were consistent across the eight clades (conditional and marginal *R* ^2^ values were 38% and 37%, respectively). In addition, by including clade in our models as a main effect, we did not see very different patterns across clades (Figure 3 panels b‐i and S4). Although clade was significant as a main effect in both models (Tables S6 and S7) indicating that overlap differed between clades, there were no significant two or three‐way interactions with clade. Across all clades, migratory species consistently experienced lower overlap between the seasons than resident species (Figure S6). See supplementary results for full details. All results were qualitatively similar when we controlled for the effects of phylogeny below the clade level using PGLS (details in Table S8 and Figure S5).

## 4 DISCUSSION

We found that support for climatic niche tracking in migrants varies depending on a number of specific factors. Support for niche tracking in migrants depended on the direction of migration (i.e., whether birds move away from breeding or nonbreeding range); the perspective in which the question is examined (i.e., from that of an individual migrant versus comparing migratory to resident species); and confounding factors such as breeding location and range size. Partly in accordance with prediction one, we found that both tropical and nontropical migratory species tracked their climatic niche between seasons if species were compared to a hypothetical situation where they did not migrate, but only when moving away from the breeding ranges. In contrast to prediction two, we found that migratory species tracked their seasonal niches to a much lower degree than resident species within the same clade. Overall, migratory birds exhibited very low overlap between seasons with 83% of migratory species having a seasonal niche overlap of less than 0.5, suggesting that migratory birds are not moving primarily to track specific climatic conditions. This overall low level of overlap between climatic niches is consistent with recent studies that report little evidence of niche tracking in migratory birds (Dufour et al., 2020; Ponti et al., 2020).

Although we found some evidence supporting our first prediction, migrants never tracked niches perfectly. As such, our results support the view that migratory species might track factors correlated with climate, and migration did not simply evolve to track climatic niches (Salewski & Bruderer, 2007; Thorup et al., 2017). From the perspective of migratory species, we found evidence that they were tracking climatic niches to some degree, at least when they moved away from their breeding range. However, in comparison with resident species there was no evidence of niche tracking in migrants, inconsistent with the findings of Gómez et al. (2016). This result is predominantly driven by the fact that despite staying in the same location year‐round, resident species inhabited very similar conditions in each season, possibly indicating quite broad but similar climatic niches in each season. Alternatively, it could indicate that even outside the tropics, the geographic distribution of resident species may be placed to experience as little climatic seasonality as possible. This is consistent with the overall pattern that there are relatively more migratory than resident species breeding outside the tropics than within the tropics and that the richness of migratory species is higher in more seasonal environments (Somveille, Manica, Butchart, & Rodrigues, 2013; Somveille et al., 2015).

The evidence for niche tracking regarding our first prediction was found to be asymmetric, indicating that the drivers for migration may be different depending on direction. Migration away from the breeding range significantly increased seasonal climatic niche overlap but migration away from the nonbreeding range did not, and in the tropics actually led to a significant reduction in niche overlap. Climate or factors correlated with climate are therefore likely to drive movement away from the breeding site, for example, a decrease in available resources in the nonbreeding season in temperate regions (Somveille et al., 2015). In contrast, the drivers for migration away from the nonbreeding range seem likely to be factors other than climate, such as seeking lower nest predation (McKinnon et al., 2010), or higher availability of nesting sites (Cox, 1968). Asymmetries have been found in previous studies which have tried to predict one season's niche from the other, and are actually expected under some hypotheses of evolution of migration (Salewski & Bruderer, 2007; Winger et al., 2019). For example, Martinez‐Meyer et al. (2004) found that the breeding niche can be predicted from the nonbreeding niche but not vice versa in the *Passerina* buntings, while Nakazawa and Peterson (2004) observed this asymmetry occurring in both directions for Nearctic–Neotropical migratory species.

In relation to both predictions tested here, the degree of niche tracking was found to differ significantly depending on the location of the breeding range, suggesting that there might be different drivers for migration operating in the tropics and outside of the tropics. For migratory species breeding in the tropics, we found no evidence for seasonal climatic niche tracking, suggesting that migration here is driven by factors other than climate, for example, by local weather aspects not captured well in our climate datasets (Reside, VanDerWal, Kutt, & Perkins, 2010). Biotic interactions such as competition and predation could be much more important for determining species distributions than the abiotic environment in the tropics (Faaborq et al., 2010; Schemske, Mittelbach, Cornell, Sobel, & Roy, 2009). However, some of the difference could be attributable to spatial biases in data quality: As lower‐quality distribution data are expected in the tropics, especially for migratory species niche overlap may be systematically underestimated there (Meyer et al., 2015; Yesson et al., 2007).

The existence of these regional differences and asymmetries could have implications for the southern‐home versus northern‐home hypotheses for the evolution of migration (Salewski & Bruderer, 2007). Our results are somewhat consistent with expectations for both theories. As the southern‐home hypothesis suggests that migrants originated in the tropics and shifted their breeding range outside to reduce predation and take advantage of high seasonal resource availability, climate would not be expected to drive migration away from the tropics. The northern‐home hypothesis in contrast proposes that species originated in temperate regions and shifted their nonbreeding range into the tropics to avoid seasonally harsh climatic conditions. Species would therefore be expected to migrate and increase seasonal niche overlap rather than to stay in the temperate breeding range all year. However, no climatic advantage to moving away from the nonbreeding range would be expected under the northern‐home hypothesis. Our results therefore support both of these theories, matching a recent biogeographic analysis across all avian species that found support for each hypothesis in different lineages (Dufour et al., 2020).

Our results were not always consistent with previous niche‐tracking studies. Overall, we found less evidence of seasonal niche tracking in migratory birds than Zurell et al. (2018), who examined Northern Hemisphere long‐distance migrants, but more evidence than Boucher‐Lalonde et al. (2013) who studied migratory and resident species across the New World. We give six possible explanations for this lack of consistency with previous studies. First, as previously discussed we found that the support for niche tracking in migrants varied depending on the perspective taken to test it (see above and Table 1). Second, as we found that niche tracking was found to vary depending on breeding location, previous studies looking at species in different geographic regions or not accounting for this geographic effect could have produced varying results. In addition, north and south hemispheres and boreal versus austral migration might systematically differ, a potentially important geographic effect not directly addressed in this study. In order to reach relatively climatically stable tropical regions, austral migrants generally have to cross less difficult geographical and ecological barriers than boreal migrants, perhaps making niche tracking more likely to drive austral migration through continuous expansion of nonbreeding areas toward the tropics. As one of the first studies finding niche tracking examined an austral migrant (Swainson's flycatcher) (Joseph & Stockwell, 2000), this is worthy of further investigation. Perhaps supporting this idea, our analyses showed that migrants achieved a similar degree of seasonal niche overlap to residents in the Xolmiini (a clade with multiple austral migrants), in contrast to most other clades (where boreal migrants prevail, Figure 3i).

As a third potential explanation for mismatch with previous niche‐tracking studies, we did not investigate physiology, which might affect species’ ability to track climatic conditions. For example, as flight is more energetically costly with increasing body size, larger birds might be expected to track climate to a lesser degree than small birds (Alerstam, Hedenstrom, & Akesson, 2003). Zurell et al. (2018) found that traits and in particular body mass explained 12%–18% of variance in tracking of niches, but we did not observe a significant clade effect (which would indicate strong influence of phylogenetically conserved traits such as body mass). As we focus only on passerine species, our study species do not exhibit as great a variation in body mass as those included in Zurell et al. (2018). Fourth, we found a significant positive relationship between range size and our niche metrics, consistent with the findings of Zurell et al. (2018). Prior to that study, range size has not been controlled for when testing niche overlap across resident and migratory species, and we show it is important to consider as otherwise differences among resident and migratory species may just reflect the differences in range size of species being studied. This is likely a reason why our results conflict with those of (Gómez et al., 2016) even when we examine the same family (Setophaga, Figure 3f). Fifth, the degree of niche overlap has been shown to vary depending on which aspects of the climatic (or broader ecological) niche are included in the analysis. For example, Zurell et al. (2018) found that migratory birds track green vegetation (measured by NDVI) to a greater degree than climatic conditions. In contrast, Dufour et al. (2020) found no evidence of niche tracking when examining the temperature niche.

Finally, differences in our results with previous studies may have arisen through methodological differences. Although highly standardized, the overlap metrics from Broennimann et al. (2012) are highly sensitive to whether climatic space is gridded for individual species separately or across the entire clade. Differences might also be attributed to data quality. Here, we used more highly resolved breeding season occurrence data and new range maps for the nonbreeding season which were compiled specifically. However, range maps are more likely to overestimate the species ranges, and consequently the niche, than point occurrence data (Eyres et al., 2017; Graham & Hijmans, 2006; Hurlbert & White, 2005). In addition, we classified migrants from descriptions of movement behavior independently of range map data. Consequently, not all species classified as migratory in our dataset had seasonal range maps, leading to possibly systematically lower seasonal overlap in climatic niches than in previous studies that focussed only on migratory species with distinct seasonal ranges.

## 5 IMPLICATIONS AND CONCLUSIONS

Our results suggest that the drivers of migration might vary across different regions and between departure from breeding and nonbreeding ranges, and offer some explanation as to the variable results of previous studies. Overall, we found relatively little support for seasonal climatic niche tracking. Despite some evidence that migratory species which breed outside of the tropics leave the breeding range to track climatic conditions, seasonal niche overlap values were overall relatively low and the niche occupied by migrants was never identical between seasons. As such, for accurate quantification of the climatic niches of birds it is essential to take into account the conditions they experience in both seasons. Finally, as migrants do not achieve the same levels of seasonal overlap as resident species, we suggest that resident species’ ranges are generally placed in less seasonal regions than migratory species. This warrants further investigation using more highly resolved distribution data such as point records (Eyres et al., 2017), particularly to understand why some species are partially migratory, with some individuals moving and others remaining in the same region year‐round (Fandos & Tellería, 2020; Fiedler, 2005; Jahn, Levey, Hostetler, & Mamani, 2010).

## CONFLICT OF INTEREST

None declared.

## AUTHOR CONTRIBUTIONS

**Alison Eyres:** Conceptualization (equal); data curation (equal); formal analysis (lead); methodology (lead); visualization (lead); writing–original draft (lead); writing–review and editing (lead). **Katrin Bohning‐Gaese:** Conceptualization (equal); data curation (equal); formal analysis (supporting); funding acquisition (supporting); supervision (supporting); writing–original draft (supporting); writing–review and editing (supporting). **David Orme:** Data curation (equal). **Carsten Rahbek:** Data curation (equal). **Susanne Fritz:** Conceptualization (lead); data curation (equal); formal analysis (supporting); funding acquisition (lead); methodology (supporting); supervision (lead); visualization (supporting); writing–original draft (supporting); writing–review and editing (supporting).

## Supporting information

Table S1

Appendix S1

## Data Availability

Processed datasets available on dryad https://doi.org/10.5061/dryad.m905qftzp.
