# Supplementary material for: A tale of two seasons: The link between seasonal migration and climatic niches in passerine birds
Source: Ecol Evol. 2020 Oct 20;10(21):11983–97. doi: 10.1002/ece3.6729 (PMC7663971; doi:10.1002/ece3.6729)
Supplement: Supplementary file 1 — Table S1 [file ECE3-10-11983-s001.docx]

Supplementary material

**Supplementary table 1.** Details for all species within our 8 clades. Migratory categorization follows Eyres et al. (2017). Range size is total terrestrial range (breeding plus non-breeding). Peak breeding months are shown and source of information denoted. For species included in our study we report climatic niche values. Reason for exclusion from analyses is noted in final column.

| **Clade** | **Species** | **Migratory behaviour** | **Breeding location** | **Range size (Km^2^)** | **Peak breeding months** | | | **Breeding source**  **(unless stated information comes from species description in handbook of birds of the world)** | **Annual niche breadth** | **Overlap** | **Overlap if**  **stay in**  **winter range** | **Overlap if stay in breeding range** | **Reason for exclusion** |
| --- | --- | --- | --- | --- | --- | --- | --- | --- | --- | --- | --- | --- | --- |
| Xolmiini | Agriornis_albicauda | resident | Tropical | 672138 | May | June | July |  | 7.71 | 0.349 | NA | NA |  |
| Xolmiini | Agriornis_lividus | resident | Non-tropical | 661396 | October | November | December |  | 7.83 | 0.104 | NA | NA |  |
| Xolmiini | Agriornis_micropterus | directional migratory | Tropical | 3197380 | October | November | December |  | 8.12 | 0.268 | 0.304 | 0.330 |  |
| Xolmiini | Agriornis_montanus | directional migratory | Tropical | 2178027 | November | December | January |  | 8.07 | 0.360 | 0.340 | 0.344 |  |
| Xolmiini | Agriornis_murinus | directional migratory | Tropical | 2335986 | October | November | December |  | 7.84 | 0.022 | 0.096 | 0.020 |  |
| Hirundinidae | Alopochelidon_fucata | directional migratory | Tropical | 3865948 | September | October | November |  | 6.32 | 0.431 | 0.434 | 0.451 |  |
| Cardinalidae | Amaurospiza_carrizalensis | resident | Non-tropical | 12206 | April | August | September | Subspecies of Amaurospiza_moesta | NA | NA | NA | NA | Small range |
| Cardinalidae | Amaurospiza_concolor | resident | Tropical | 729710 | May | June | July |  | 6.96 | 0.190 | NA | NA |  |
| Cardinalidae | Amaurospiza_moesta | resident | Tropical | 638684 | April | August | September |  | 6.11 | 0.277 | NA | NA |  |
| Hirundinidae | Atticora_fasciata | resident | Tropical | 7141627 | February | March | April |  | 6.42 | 0.602 | NA | NA |  |
| Hirundinidae | Atticora_melanoleuca | resident | Tropical | 2598019 | February | March | April |  | 6.22 | 0.465 | NA | NA |  |
| Setophaga | Basileuterus_basilicus | resident | Non-tropical | 12108 | April | May | June |  | NA | NA | NA | NA | Small range |
| Setophaga | Basileuterus_belli | resident | Non-tropical | 667160 | April | May | June |  | 6.76 | 0.237 | NA | NA |  |
| Setophaga | Basileuterus_culicivorus | resident | Tropical | 7031509 | April | May | June |  | 7.26 | 0.624 | NA | NA |  |
| Setophaga | Basileuterus_ignotus | resident | Non-tropical | 8538 | May | June | July |  | NA | NA | NA | NA | Small range |
| Setophaga | Basileuterus_lachrymosus | directional migratory | Non-tropical | 742090 | April | May | June | Close relative (Basileuterus rufifrons) | 6.80 | 0.254 | 0.305 | 0.258 |  |
| Setophaga | Basileuterus_melanogenys | resident | Non-tropical | 45398 | April | May | June |  | 6.03 | 0.356 | NA | NA |  |
| Setophaga | Basileuterus_rufifrons | resident | Non-tropical | 1400516 | April | May | June |  | 7.26 | 0.227 | NA | NA |  |
| Setophaga | Basileuterus_trifasciatus | resident | Non-tropical | 85173 | April | May | June |  | 5.42 | 0.488 | NA | NA |  |
| Setophaga | Basileuterus_tristriatus | resident | Tropical | 830395 | April | May | June |  | 6.81 | 0.527 | NA | NA |  |
| Oenanthe | Campicoloides_bifasciatus | resident | Tropical | 361313 | September | October | November |  | 5.43 | 0.465 | NA | NA |  |
| Setophaga | Cardellina_canadensis | directional migratory | Tropical | 5761791 | May | June | July |  | 6.83 | 0.172 | 0.519 | 0.001 |  |
| Setophaga | Cardellina_pusilla | directional migratory | Non-tropical | 10272546 | May | June | July |  | 6.83 | 0.327 | 0.259 | 0.043 |  |
| Setophaga | Cardellina_rubra | resident | Non-tropical | 450795 | May | June | July |  | 6.73 | 0.017 | NA | NA |  |
| Setophaga | Cardellina_rubrifrons | directional migratory | Non-tropical | 1064900 | May | June | July |  | 6.96 | 0.004 | 0.092 | 0.001 |  |
| Setophaga | Cardellina_versicolor | resident | Non-tropical | 128599 | April | May | June |  | 6.37 | 0.334 | NA | NA |  |
| Cardinalidae | Cardinalis_cardinalis | resident | Non-tropical | 6011247 | February | March | April |  | 7.72 | 0.384 | NA | NA |  |
| Cardinalidae | Cardinalis_phoeniceus | resident | Non-tropical | 90334 | June | July | August |  | 5.62 | 0.192 | NA | NA |  |
| Cardinalidae | Cardinalis_sinuatus | resident | Non-tropical | 1744390 | May | June | July |  | 7.57 | 0.003 | NA | NA |  |
| Cardinalidae | Caryothraustes_canadensis | resident | Tropical | 3200849 | February | March | April |  | 6.92 | 0.528 | NA | NA |  |
| Cardinalidae | Caryothraustes_poliogaster | resident | Non-tropical | 381225 | April | May | June |  | 6.74 | 0.426 | NA | NA |  |
| Setophaga | Catharopeza_bishopi | resident | Non-tropical | 524 | May | June | July |  | NA | NA | NA | NA | Small range |
| Hirundinidae | Cecropis_abyssinica | directional migratory | Tropical | 13491417 | March | May | June |  | 7.00 | 0.780 | 0.813 | 0.800 |  |
| Hirundinidae | Cecropis_badia | resident | Non-tropical | 185565 | May | June | July |  | 5.55 | 0.317 | NA | NA |  |
| Hirundinidae | Cecropis_cucullata | directional migratory | Tropical | 3930206 | October | November | December |  | 6.14 | 0.418 | 0.291 | 0.227 |  |
| Hirundinidae | Cecropis_daurica | directional migratory | Tropical | 23223171 | May | June | July |  | 7.38 | 0.369 | 0.173 | 0.161 |  |
| Hirundinidae | Cecropis_domicella | resident | Non-tropical | 2556348 | April | May | June |  | 6.49 | 0.508 | NA | NA |  |
| Hirundinidae | Cecropis_hyperythra | resident | Non-tropical | 66111 | June | July | August |  | 5.29 | 0.226 | NA | NA |  |
| Hirundinidae | Cecropis_semirufa | directional migratory | Tropical | 9859524 | April | August | December |  | 7.00 | 0.530 | 0.669 | 0.735 |  |
| Hirundinidae | Cecropis_senegalensis | dispersive migratory | Tropical | 13042941 | April | August | December |  | NA | NA | NA | NA | Migratory status |
| Hirundinidae | Cecropis_striolata | directional migratory | Tropical | 2350838 | April | May | June |  | 6.81 | 0.355 | 0.383 | 0.334 |  |
| Hirundinidae | Cheramoeca_leucosterna | resident | Tropical | 3383270 | September | October | November |  | 6.58 | 0.592 | NA | NA |  |
| Cardinalidae | Chlorothraupis_carmioli | resident | Non-tropical | 135461 | March | April | May |  | 6.07 | 0.122 | NA | NA |  |
| Cardinalidae | Chlorothraupis_frenata | resident | Tropical | 372800 | January | February | March | Close relative (Chlorothraupis stolzmanni) | 6.72 | 0.230 | NA | NA |  |
| Cardinalidae | Chlorothraupis_olivacea | resident | Non-tropical | 208277 | January | February | March | Close relative (Chlorothraupis stolzmanni) | 6.22 | 0.160 | NA | NA |  |
| Cardinalidae | Chlorothraupis_stolzmanni | resident | Tropical | 171925 | January | February | March |  | 6.09 | 0.417 | NA | NA |  |
| Xolmiini | Cnemarchus_erythropygius | resident | Tropical | 255508 | October | November | December |  | 6.87 | 0.426 | NA | NA |  |
| Corvus | Coloeus_dauuricus | directional migratory | Non-tropical | 7216488 | March | April | May |  | 6.68 | 0.262 | 0.484 | 0.432 |  |
| Corvus | Coloeus_monedula | directional migratory | Non-tropical | 18477193 | April | May | June |  | 7.22 | 0.325 | 0.280 | 0.247 |  |
| Corvus | Corvus_albicollis | resident | Tropical | 2939935 | September | October | November |  | 6.28 | 0.544 | NA | NA |  |
| Corvus | Corvus_albus | resident | Tropical | 19450758 | April | June | September |  | 7.22 | 0.806 | NA | NA |  |
| Corvus | Corvus_bennetti | nomadic | Tropical | 4702793 | July | August | September |  | NA | NA | NA | NA | Migratory status |
| Corvus | Corvus_brachyrhynchos | directional migratory | Non-tropical | 11655107 | March | April | May |  | 6.98 | 0.596 | 0.686 | 0.632 |  |
| Corvus | Corvus_capensis | resident | Tropical | 5152953 | September | October | December |  | 6.40 | 0.733 | NA | NA |  |
| Corvus | Corvus_caurinus | resident | Non-tropical | 578013 | April | May | June |  | 6.08 | 0.016 | NA | NA |  |
| Corvus | Corvus_corax | dispersive migratory | Non-tropical | 52289434 | February | March | April |  | NA | NA | NA | NA | Migratory status |
| Corvus | Corvus_cornix | directional migratory | Non-tropical | 18221353 | February | March | April |  | 7.36 | 0.339 | 0.382 | 0.485 |  |
| Corvus | Corvus_corone | directional migratory | Non-tropical | 19360797 | April | May | June |  | 7.49 | 0.411 | 0.344 | 0.350 |  |
| Corvus | Corvus_coronoides | resident | Tropical | 4181741 | July | August | September |  | 7.02 | 0.321 | NA | NA |  |
| Corvus | Corvus_crassirostris | resident | Non-tropical | 676412 | January | February | March |  | 6.40 | 0.120 | NA | NA |  |
| Corvus | Corvus_cryptoleucus | dispersive migratory | Non-tropical | 1903634 | April | May | June |  | NA | NA | NA | NA | Migratory status |
| Corvus | Corvus_culminatus | resident | Non-tropical | 2641822 | May | June | July |  | 7.16 | 0.099 | NA | NA |  |
| Corvus | Corvus_edithae | resident | Tropical | 1463196 | February | May | June |  | 5.26 | 0.762 | NA | NA |  |
| Corvus | Corvus_enca | resident | Tropical | 1761542 | May | June | July |  | 5.67 | 0.509 | NA | NA |  |
| Corvus | Corvus_florensis | resident | Non-tropical | 15363 | October | November | January |  | 5.55 | 0.081 | NA | NA |  |
| Corvus | Corvus_frugilegus | directional migratory | Non-tropical | 22423121 | March | April | May |  | 7.15 | 0.554 | 0.642 | 0.661 |  |
| Corvus | Corvus_fuscicapillus | resident | Tropical | 101915 | July | August | September | Close relative (Corvus tristis) | 4.60 | 0.385 | NA | NA |  |
| Corvus | Corvus_hawaiiensis | resident | Non-tropical | 8885 | March | April | May |  | NA | NA | NA | NA | Extinct |
| Corvus | Corvus_imparatus | resident | Non-tropical | 203068 | April | May | June |  | 5.37 | 0.039 | NA | NA |  |
| Corvus | Corvus_insularis | resident | Tropical | 44623 | February | March | April |  | 4.86 | 0.244 | NA | NA |  |
| Corvus | Corvus_jamaicensis | resident | Non-tropical | 11021 | May | June | July | Gosse. The birds of Jamaica | NA | NA | NA | NA | Small range |
| Corvus | Corvus_kubaryi | resident | Non-tropical | 696 | September | November | January |  | NA | NA | NA | NA | Small range |
| Corvus | Corvus_leucognaphalus | resident | Non-tropical | 58315 | March | April | May |  | 5.10 | 0.418 | NA | NA |  |
| Corvus | Corvus_levaillantii | resident | Non-tropical | 1660244 | January | February | March |  | 6.95 | 0.014 | NA | NA |  |
| Corvus | Corvus_macrorhynchos | resident | Tropical | 11333373 | March | July | November |  | 7.32 | 0.716 | NA | NA |  |
| Corvus | Corvus_meeki | resident | Non-tropical | 9566 | August | September | October |  | NA | NA | NA | NA | Small range |
| Corvus | Corvus_mellori | dispersive migratory | Non-tropical | 1130055 | August | September | October |  | NA | NA | NA | NA | Migratory status |
| Corvus | Corvus_minutus | resident | Non-tropical | 17088 | April | May | June |  | 5.23 | 0.027 | NA | NA |  |
| Corvus | Corvus_moneduloides | resident | Tropical | 16647 | October | November | December |  | 4.26 | 0 | NA | NA |  |
| Corvus | Corvus_nasicus | resident | Non-tropical | 52771 | April | May | June |  | 4.98 | 0.024 | NA | NA |  |
| Corvus | Corvus_orru | resident | Tropical | 5025206 | January | April | November |  | 7.13 | 0.428 | NA | NA |  |
| Corvus | Corvus_ossifragus | directional migratory | Non-tropical | 1484475 | March | April | May |  | 5.35 | 0.507 | 0.507 | 0.507 |  |
| Corvus | Corvus_palmarum | resident | Non-tropical | 58206 | April | May | June |  | 5.14 | 0.288 | NA | NA |  |
| Corvus | Corvus_rhipidurus | resident | Tropical | 3534669 | May | June | September |  | 6.65 | 0.450 | NA | NA |  |
| Corvus | Corvus_ruficollis | directional migratory | Non-tropical | 13495906 | February | March | April |  | 6.76 | 0.547 | 0.547 | 0.547 |  |
| Corvus | Corvus_sinaloae | resident | Non-tropical | 113373 | May | June | July |  | 5.15 | 0.001 | NA | NA |  |
| Corvus | Corvus_splendens | resident | Tropical | 4610234 | April | May | June |  | 7.11 | 0.127 | NA | NA |  |
| Corvus | Corvus_tasmanicus | resident | Non-tropical | 226760 | July | August | September |  | 6.15 | 0.056 | NA | NA |  |
| Corvus | Corvus_torquatus | directional migratory | Non-tropical | 3080116 | February | March | April |  | 6.38 | 0.375 | 0.375 | 0.375 |  |
| Corvus | Corvus_tristis | resident | Tropical | 679644 | July | August | September |  | 5.91 | 0.403 | NA | NA |  |
| Corvus | Corvus_typicus | resident | Tropical | 125938 | May | June | July | Close relative (Corvus enca) | 5.05 | 0.614 | NA | NA |  |
| Corvus | Corvus_unicolor | resident | Tropical | 7108 | April | May | June |  | NA | NA | NA | NA | Small range |
| Corvus | Corvus_validus | resident | Tropical | 26571 | May | June | July | Close relative (Corvus enca) | 4.20 | 0.291 | NA | NA |  |
| Corvus | Corvus_violaceus | resident | Tropical | 27753 | May | June | July |  | 4.78 | 0.051 | NA | NA |  |
| Corvus | Corvus_woodfordi | resident | Non-tropical | 14820 | July | August | September |  | NA | NA | NA | NA | Small range |
| Cardinalidae | Cyanocompsa_brissonii | resident | Tropical | 5750072 | November | December | January |  | 7.27 | 0.234 | NA | NA |  |
| Cardinalidae | Cyanocompsa_cyanoides | resident | Tropical | 7473561 | February | March | April |  | 7.02 | 0.652 | NA | NA |  |
| Cardinalidae | Cyanocompsa_parellina | resident | Non-tropical | 845900 | April | May | June |  | 6.90 | 0.375 | NA | NA |  |
| Cardinalidae | Cyanoloxia_glaucocaerulea | directional migratory | Tropical | 1307461 | October | November | December |  | 6.52 | 0.217 | 0.213 | 0.208 |  |
| Vireonidae | Cyclarhis_gujanensis | resident | Tropical | 13369924 | May | June | July |  | NA | NA | NA | NA | Missed out accidentally |
| Vireonidae | Cyclarhis_nigrirostris | resident | Tropical | 280930 | May | June | July |  | 6.24 | 0.531 | NA | NA |  |
| Hirundinidae | Delichon_dasypus | directional migratory | Tropical | 7134509 | May | June | July |  | 6.88 | 0.387 | 0.371 | 0.188 |  |
| Hirundinidae | Delichon_nipalense | resident | Non-tropical | 952036 | May | June | July |  | 6.97 | 0.087 | NA | NA |  |
| Hirundinidae | Delichon_urbicum | directional migratory | Tropical | 40644043 | May | June | July |  | 6.87 | 0.367 | 0.404 | 0.124 |  |
| Oenanthe | Emarginata_schlegelii | resident | Tropical | 991696 | September | October | November |  | 5.81 | 0.611 | NA | NA |  |
| Oenanthe | Emarginata_sinuata | resident | Non-tropical | 868832 | October | November | December |  | 5.68 | 0.246 | NA | NA |  |
| Oenanthe | Emarginata_tractrac | resident | Tropical | 841312 | September | October | November |  | 5.76 | 0.568 | NA | NA |  |
| Cardinalidae | Granatellus_pelzelni | resident | Tropical | 4351514 | February | March | April |  | 6.94 | 0.361 | NA | NA |  |
| Cardinalidae | Granatellus_sallaei | resident | Non-tropical | 398666 | June | July | August | Close relative (Granatellus venustus) | 6.30 | 0.034 | NA | NA |  |
| Cardinalidae | Granatellus_venustus | resident | Non-tropical | 312066 | June | July | August |  | 6.82 | 0.006 | NA | NA |  |
| Cardinalidae | Habia_atrimaxillaris | resident | Non-tropical | 10765 | February | March | April |  | NA | NA | NA | NA | Small range |
| Cardinalidae | Habia_cristata | resident | Non-tropical | 72488 | May | June | July |  | 5.56 | 0.492 | NA | NA |  |
| Cardinalidae | Habia_fuscicauda | resident | Non-tropical | 879196 | April | May | June |  | 6.57 | 0.255 | NA | NA |  |
| Cardinalidae | Habia_gutturalis | resident | Non-tropical | 109785 | February | March | April |  | 5.99 | 0.120 | NA | NA |  |
| Cardinalidae | Habia_rubica | resident | Tropical | 6296347 | April | May | June |  | 7.17 | 0.612 | NA | NA |  |
| Hirundinidae | Haplochelidon_andecola | resident | Tropical | 594379 | November | December | January |  | 5.68 | 0.245 | NA | NA |  |
| Xolmiini | Heteroxolmis_dominicana | resident | Tropical | 1193268 | October | November | December |  | 6.93 | 0.186 | NA | NA |  |
| Hirundinidae | Hirundo_aethiopica | dispersive migratory | Tropical | 5319088 | April | June | August |  | NA | NA | NA | NA | Migratory status |
| Hirundinidae | Hirundo_albigularis | directional migratory | Tropical | 4553252 | October | November | December |  | 6.34 | 0.276 | 0.256 | 0.249 |  |
| Hirundinidae | Hirundo_angolensis | resident | Tropical | 2219782 | April | August | December |  | 6.30 | 0.799 | NA | NA |  |
| Hirundinidae | Hirundo_atrocaerulea | directional migratory | Tropical | 509541 | November | December | January |  | 5.71 | 0.330 | 0.466 | 0.037 |  |
| Hirundinidae | Hirundo_dimidiata | directional migratory | Tropical | 3365705 | August | September | October |  | 6.46 | 0.276 | 0.249 | 0.353 |  |
| Hirundinidae | Hirundo_domicola | resident | Non-tropical | 135795 | March | April | May |  | 6.05 | 0.217 | NA | NA |  |
| Hirundinidae | Hirundo_leucosoma | dispersive migratory | Non-tropical | 1478308 | April | May | June |  | NA | NA | NA | NA | Migratory status |
| Hirundinidae | Hirundo_lucida | resident | Tropical | 2706831 | August | September | October |  | 6.90 | 0.163 | NA | NA |  |
| Hirundinidae | Hirundo_megaensis | resident | Non-tropical | 61338 | April | May | June |  | 5.16 | 0.469 | NA | NA |  |
| Hirundinidae | Hirundo_neoxena | directional migratory | Tropical | 4596354 | August | September | October |  | 7.19 | 0.689 | 0.685 | 0.690 |  |
| Hirundinidae | Hirundo_nigrita | resident | Tropical | 3410894 | January | July | December |  | 6.32 | 0.832 | NA | NA |  |
| Hirundinidae | Hirundo_nigrorufa | resident | Tropical | 760081 | July | August | September |  | 6.13 | 0 | NA | NA |  |
| Hirundinidae | Hirundo_rustica | directional migratory | Tropical | 95573950 | May | June | July |  | 7.33 | 0.549 | 0.397 | 0.185 |  |
| Hirundinidae | Hirundo_smithii | directional migratory | Tropical | 15916287 | April | May | October |  | 6.94 | 0.758 | 0.827 | 0.741 |  |
| Hirundinidae | Hirundo_tahitica | resident | Tropical | 3585857 | April | May | June |  | 6.29 | 0.715 | NA | NA |  |
| Vireonidae | Hylophilus_amaurocephalus | resident | Tropical | 1144644 | December | January | February |  | 6.96 | 0.107 | NA | NA |  |
| Vireonidae | Hylophilus_aurantiifrons | resident | Non-tropical | 634104 | July | August | September |  | 6.54 | 0.079 | NA | NA |  |
| Vireonidae | Hylophilus_brunneiceps | resident | Tropical | 762523 | March | May | July | Close relative (Hylophilus muscicapinus) | 6.19 | 0.264 | NA | NA |  |
| Vireonidae | Hylophilus_decurtatus | resident | Tropical | 1092938 | March | April | May |  | 6.68 | 0.293 | NA | NA |  |
| Vireonidae | Hylophilus_flavipes | resident | Non-tropical | 989357 | April | May | June |  | 6.69 | 0.359 | NA | NA |  |
| Vireonidae | Hylophilus_hypoxanthus | resident | Tropical | 3726094 | March | May | July | Close relative (Hylophilus pectoralis) | 6.84 | 0.595 | NA | NA |  |
| Vireonidae | Hylophilus_muscicapinus | resident | Tropical | 2757069 | February | March | April |  | 6.94 | 0.556 | NA | NA |  |
| Vireonidae | Hylophilus_ochraceiceps | resident | Tropical | 6171373 | April | May | June |  | 6.99 | 0.711 | NA | NA |  |
| Vireonidae | Hylophilus_olivaceus | resident | Tropical | 171247 | May | June | July |  | 6.22 | 0.359 | NA | NA |  |
| Vireonidae | Hylophilus_pectoralis | resident | Tropical | 2706524 | March | May | July |  | 7.10 | 0.531 | NA | NA |  |
| Vireonidae | Hylophilus_poicilotis | resident | Tropical | 862356 | August | September | October |  | 6.16 | 0.595 | NA | NA |  |
| Vireonidae | Hylophilus_sclateri | resident | Non-tropical | 331096 | March | May | July | Close relative (Hylophilus pectoralis) | 5.90 | 0.188 | NA | NA |  |
| Vireonidae | Hylophilus_semibrunneus | resident | Tropical | 241088 | April | May | June |  | 6.04 | 0.517 | NA | NA |  |
| Vireonidae | Hylophilus_semicinereus | resident | Tropical | 4186330 | March | May | July | Close relative (Hylophilus pectoralis) | 6.91 | 0.638 | NA | NA |  |
| Vireonidae | Hylophilus_thoracicus | resident | Tropical | 3785961 | March | May | July | Close relative (Hylophilus pectoralis) | 7.09 | 0.665 | NA | NA |  |
| Xolmiini | Hymenops_perspicillatus | directional migratory | Tropical | 4359166 | November | December | January |  | 8.25 | 0.041 | 0.059 | 0.005 |  |
| Xolmiini | Knipolegus_aterrimus | directional migratory | Tropical | 2454073 | November | December | January |  | 8.08 | 0.255 | 0.198 | 0.212 |  |
| Xolmiini | Knipolegus_cyanirostris | directional migratory | Tropical | 2456907 | November | December | January |  | 7.06 | 0.139 | 0.138 | 0.071 |  |
| Xolmiini | Knipolegus_franciscanus | resident | Tropical | 176899 | November | December | January |  | 6.43 | 0 | NA | NA |  |
| Xolmiini | Knipolegus_hudsoni | directional migratory | Tropical | 2158173 | November | December | January | Close relative (Knipolgus Aterrimus) | 7.63 | 0 | 0.034 | 0.000 |  |
| Xolmiini | Knipolegus_lophotes | resident | Tropical | 2550391 | September | October | November |  | 6.87 | 0.697 | NA | NA |  |
| Xolmiini | Knipolegus_nigerrimus | resident | Tropical | 740470 | September | October | November | Close relative (Knipolgus lophotes) | 6.58 | 0.806 | NA | NA |  |
| Xolmiini | Knipolegus_orenocensis | resident | Tropical | 1112688 | February | March | April |  | 6.88 | 0.286 | NA | NA |  |
| Xolmiini | Knipolegus_poecilocercus | resident | Tropical | 2440993 | July | August | September |  | 6.95 | 0.489 | NA | NA |  |
| Xolmiini | Knipolegus_poecilurus | resident | Tropical | 1011567 | April | June | August |  | 7.27 | 0.501 | NA | NA |  |
| Xolmiini | Knipolegus_signatus | resident | Tropical | 433717 | October | December | January |  | 7.71 | 0.382 | NA | NA |  |
| Xolmiini | Knipolegus_striaticeps | resident | Tropical | 1315406 | November | December | January |  | 7.44 | 0.035 | NA | NA |  |
| Xolmiini | Lessonia_oreas | resident | Tropical | 1035068 | October | November | December |  | 7.46 | 0.527 | NA | NA |  |
| Xolmiini | Lessonia_rufa | directional migratory | Tropical | 3376010 | September | October | November |  | 7.86 | 0.214 | 0.504 | 0.506 |  |
| Oenanthe | Monticola_angolensis | resident | Tropical | 2443581 | September | October | November |  | 6.34 | 0.380 | NA | NA |  |
| Oenanthe | Monticola_brevipes | resident | Tropical | 1333796 | November | December | January |  | 6.02 | 0.047 | NA | NA |  |
| Oenanthe | Monticola_cinclorhynchus | directional migratory | Non-tropical | 1134300 | May | June | July |  | 6.69 | 0.207 | 0.108 | 0.119 |  |
| Oenanthe | Monticola_explorator | resident | Non-tropical | 574123 | September | October | November |  | 5.54 | 0.626 | NA | NA |  |
| Oenanthe | Monticola_gularis | directional migratory | Non-tropical | 4205882 | May | June | July |  | 5.97 | 0.324 | 0.118 | 0.000 |  |
| Oenanthe | Monticola_imerina | resident | Tropical | 16383 | November | December | January |  | NA | NA | NA | NA | Small range |
| Oenanthe | Monticola_rufiventris | directional migratory | Non-tropical | 3798128 | April | May | June |  | 7.04 | 0.306 | 0.302 | 0.305 |  |
| Oenanthe | Monticola_rufocinereus | resident | Tropical | 990103 | March | May | September |  | 5.90 | 0.788 | NA | NA |  |
| Oenanthe | Monticola_rupestris | resident | Tropical | 730718 | October | November | December |  | 5.70 | 0.152 | NA | NA |  |
| Oenanthe | Monticola_saxatilis | directional migratory | Tropical | 12544719 | May | June | July |  | 6.53 | 0.596 | 0.530 | 0.035 |  |
| Oenanthe | Monticola_semirufus | resident | Non-tropical | 509587 | June | July | August |  | 6.20 | 0.066 | NA | NA |  |
| Oenanthe | Monticola_sharpei | resident | Tropical | 347795 | November | December | January |  | 5.98 | 0.025 | NA | NA |  |
| Oenanthe | Monticola_solitarius | directional migratory | Tropical | 24450543 | April | May | June |  | 7.27 | 0.585 | 0.349 | 0.313 |  |
| Xolmiini | Muscisaxicola_albifrons | resident | Tropical | 413093 | November | December | January |  | 7.22 | 0.386 | NA | NA |  |
| Xolmiini | Muscisaxicola_albilora | directional migratory | Tropical | 1689648 | October | November | December |  | 7.55 | 0.294 | 0.459 | 0.113 |  |
| Xolmiini | Muscisaxicola_alpinus | resident | Tropical | 195225 | September | October | November |  | 6.17 | 0.469 | NA | NA |  |
| Xolmiini | Muscisaxicola_capistratus | directional migratory | Tropical | 1870678 | October | November | December |  | 7.85 | 0.274 | 0.391 | 0.099 |  |
| Xolmiini | Muscisaxicola_cinereus | directional migratory | Tropical | 876904 | October | December | February |  | 7.67 | 0.430 | 0.452 | 0.394 |  |
| Xolmiini | Muscisaxicola_flavinucha | directional migratory | Tropical | 1557884 | November | December | January |  | 7.51 | 0.408 | 0.403 | 0.027 |  |
| Xolmiini | Muscisaxicola_fluviatilis | resident | Tropical | 2229594 | August | September | October |  | 7.32 | 0.295 | NA | NA |  |
| Xolmiini | Muscisaxicola_frontalis | directional migratory | Tropical | 986277 | November | January | February |  | 7.31 | 0.255 | 0.362 | 0.028 |  |
| Xolmiini | Muscisaxicola_griseus | resident | Tropical | 528512 | November | December | January |  | 7.28 | 0.360 | NA | NA |  |
| Xolmiini | Muscisaxicola_juninensis | resident | Tropical | 575418 | October | November | December |  | 7.23 | 0.477 | NA | NA |  |
| Xolmiini | Muscisaxicola_maclovianus | directional migratory | Tropical | 2968212 | September | December | February |  | 8.01 | 0.507 | 0.531 | 0.300 |  |
| Xolmiini | Muscisaxicola_maculirostris | directional migratory | Tropical | 2181603 | November | January | February |  | 7.79 | 0.411 | 0.399 | 0.349 |  |
| Xolmiini | Muscisaxicola_rufivertex | resident | Tropical | 1094826 | September | November | January |  | 7.63 | 0.591 | NA | NA |  |
| Setophaga | Myioborus_albifacies | resident | Non-tropical | 24508 | April | May | June | Close relative (Myioborus ornatus) | 5.80 | 0.012 | NA | NA |  |
| Setophaga | Myioborus_albifrons | resident | Non-tropical | 64149 | April | May | June |  | 5.58 | 0.363 | NA | NA |  |
| Setophaga | Myioborus_brunniceps | resident | Tropical | 406238 | October | November | December |  | 6.85 | 0.115 | NA | NA |  |
| Setophaga | Myioborus_cardonai | resident | Non-tropical | 12254 | April | May | June | Close relative (Myioborus ornatus) | NA | NA | NA | NA | Small range |
| Setophaga | Myioborus_castaneocapilla | resident | Non-tropical | 159507 | April | May | June | Close relative (Myioborus ornatus) | 5.95 | 0.186 | NA | NA |  |
| Setophaga | Myioborus_flavivertex | resident | Non-tropical | 13239 | April | May | June |  | NA | NA | NA | NA | Small range |
| Setophaga | Myioborus_melanocephalus | resident | Tropical | 516617 | July | September | November |  | 7.03 | 0.517 | NA | NA |  |
| Setophaga | Myioborus_miniatus | resident | Tropical | 2680254 | February | April | June |  | 7.19 | 0.572 | NA | NA |  |
| Setophaga | Myioborus_ornatus | resident | Non-tropical | 330131 | April | May | June |  | 6.11 | 0.544 | NA | NA |  |
| Setophaga | Myioborus_pariae | resident | Non-tropical | 9163 | April | May | June | Close relative (Myioborus ornatus) | NA | NA | NA | NA | Small range |
| Setophaga | Myioborus_pictus | directional migratory | Non-tropical | 1542006 | April | May | June |  | 7.10 | 0.143 | 0.275 | 0.175 |  |
| Setophaga | Myioborus_torquatus | resident | Non-tropical | 51762 | March | April | May |  | 6.01 | 0.078 | NA | NA |  |
| Xolmiini | Myiotheretes_fumigatus | resident | Tropical | 645230 | July | August | September |  | 6.85 | 0.499 | NA | NA |  |
| Xolmiini | Myiotheretes_fuscorufus | resident | Tropical | 178628 | September | October | November |  | 6.96 | 0.452 | NA | NA |  |
| Xolmiini | Myiotheretes_pernix | resident | Non-tropical | 13239 | February | April | May | Close relative (Myiotheretes striaticollis) | NA | NA | NA | NA | Small range |
| Xolmiini | Myiotheretes_striaticollis | resident | Tropical | 1117764 | February | April | May |  | 7.77 | 0.669 | NA | NA |  |
| Setophaga | Myiothlypis_bivittata | resident | Tropical | 758620 | November | December | January |  | 6.57 | 0.316 | NA | NA |  |
| Setophaga | Myiothlypis_chlorophrys | resident | Tropical | 63112 | May | June | July | Close relative (Myiothlypis fulvicauda) | 5.90 | 0.447 | NA | NA |  |
| Setophaga | Myiothlypis_chrysogaster | resident | Tropical | 84365 | May | June | July | Close relative (Myiothlypis fulvicauda) | 6.22 | 0.217 | NA | NA |  |
| Setophaga | Myiothlypis_cinereicollis | resident | Non-tropical | 133925 | April | June | July |  | 6.43 | 0.258 | NA | NA |  |
| Setophaga | Myiothlypis_conspicillata | resident | Non-tropical | 24155 | April | May | June |  | 5.55 | 0.212 | NA | NA |  |
| Setophaga | Myiothlypis_coronata | resident | Tropical | 776072 | May | June | July |  | 7.15 | 0.431 | NA | NA |  |
| Setophaga | Myiothlypis_flaveola | resident | Tropical | 4417088 | October | November | December |  | 6.90 | 0.361 | NA | NA |  |
| Setophaga | Myiothlypis_fraseri | resident | Tropical | 123262 | January | February | March |  | 6.31 | 0.158 | NA | NA |  |
| Setophaga | Myiothlypis_fulvicauda | resident | Tropical | 3151565 | May | June | July |  | 7.11 | 0.589 | NA | NA |  |
| Setophaga | Myiothlypis_griseiceps | resident | Non-tropical | 11829 | May | June | July |  | NA | NA | NA | NA | Small range |
| Setophaga | Myiothlypis_leucoblephara | resident | Tropical | 1400119 | October | November | December | Close relative (Basileuterus leucoblepharus) | 6.20 | 0.217 | NA | NA |  |
| Setophaga | Myiothlypis_leucophrys | resident | Tropical | 885842 | October | November | December |  | 6.44 | 0.062 | NA | NA |  |
| Setophaga | Myiothlypis_luteoviridis | resident | Tropical | 642119 | August | September | October |  | 6.77 | 0.390 | NA | NA |  |
| Setophaga | Myiothlypis_nigrocristata | resident | Tropical | 523292 | May | June | July |  | 6.70 | 0.416 | NA | NA |  |
| Setophaga | Myiothlypis_rivularis | resident | Tropical | 4702898 | February | March | April |  | 7.17 | 0.496 | NA | NA |  |
| Setophaga | Myiothlypis_signata | resident | Tropical | 436469 | December | January | February |  | 6.58 | 0.069 | NA | NA |  |
| Oenanthe | Myrmecocichla_aethiops | resident | Tropical | 2903618 | June | July | August |  | 6.33 | 0.071 | NA | NA |  |
| Oenanthe | Myrmecocichla_formicivora | resident | Tropical | 2396113 | October | November | December |  | 6.13 | 0.191 | NA | NA |  |
| Oenanthe | Myrmecocichla_melaena | resident | Non-tropical | 217073 | June | July | August |  | 6.00 | 0.030 | NA | NA |  |
| Oenanthe | Myrmecocichla_nigra | resident | Tropical | 4932444 | March | July | November |  | 6.70 | 0.786 | NA | NA |  |
| Oenanthe | Myrmecocichla_tholloni | resident | Tropical | 400082 | June | July | August |  | 6.20 | 0.074 | NA | NA |  |
| Hirundinidae | Neochelidon_tibialis | resident | Tropical | 4350340 | March | April | May |  | 6.42 | 0.670 | NA | NA |  |
| Xolmiini | Neoxolmis_rufiventris | directional migratory | Non-tropical | 1484896 | November | December | January |  | 7.51 | 0 | 0 | 0.000 |  |
| Turdus | Nesocichla_eremita | resident | Non-tropical | 96 | October | November | December |  | NA | NA | NA | NA | Small range |
| Hirundinidae | Notiochelidon_cyanoleuca | directional migratory | Tropical | 16832582 | March | April | May |  | 7.22 | 0.634 | 0.727 | 0.635 |  |
| Hirundinidae | Notiochelidon_flavipes | resident | Tropical | 411151 | July | August | September |  | 5.97 | 0.428 | NA | NA |  |
| Hirundinidae | Notiochelidon_murina | resident | Tropical | 975553 | August | September | October |  | 6.27 | 0.487 | NA | NA |  |
| Hirundinidae | Notiochelidon_pileata | resident | Non-tropical | 140752 | April | May | June |  | 5.94 | 0.248 | NA | NA |  |
| Oenanthe | Oenanthe_albonigra | resident | Non-tropical | 1205077 | March | April | May |  | 6.26 | 0.581 | NA | NA |  |
| Oenanthe | Oenanthe_bottae | resident | Non-tropical | 509298 | March | April | May |  | 5.85 | 0.485 | NA | NA |  |
| Oenanthe | Oenanthe_chrysopygia | directional migratory | Non-tropical | 4751993 | May | June | July |  | 6.34 | 0.173 | 0.023 | 0.034 |  |
| Oenanthe | Oenanthe_cypriaca | directional migratory | Non-tropical | 1570563 | April | May | June |  | NA | NA | NA | NA | Small range |
| Oenanthe | Oenanthe_deserti | directional migratory | Non-tropical | 21439256 | May | June | July |  | 6.44 | 0.520 | 0.133 | 0.097 |  |
| Oenanthe | Oenanthe_dubia | unknown | Non-tropical | 72896 | May | June | July |  | NA | NA | NA | NA | Migratory status |
| Oenanthe | Oenanthe_familiaris | resident | Tropical | 8163458 | March | September | December |  | 6.90 | 0.768 | NA | NA |  |
| Oenanthe | Oenanthe_finschii | directional migratory | Non-tropical | 4089050 | April | May | June |  | 6.67 | 0.504 | 0.356 | 0.230 |  |
| Oenanthe | Oenanthe_fusca | resident | Non-tropical | 1479910 | April | May | June |  | 6.30 | 0.069 | NA | NA |  |
| Oenanthe | Oenanthe_heuglini | dispersive migratory | Non-tropical | 3747670 | January | February | March |  | NA | NA | NA | NA | Migratory status |
| Oenanthe | Oenanthe_hispanica | directional migratory | Non-tropical | 8243887 | April | May | June |  | 6.38 | 0.384 | 0.417 | 0.302 |  |
| Oenanthe | Oenanthe_isabellina | directional migratory | Tropical | 23442815 | April | May | June |  | 6.55 | 0.534 | 0.322 | 0.239 |  |
| Oenanthe | Oenanthe_leucopyga | resident | Non-tropical | 5978678 | March | April | May |  | 6.30 | 0.750 | NA | NA |  |
| Oenanthe | Oenanthe_leucura | resident | Non-tropical | 1654812 | March | April | May |  | 5.94 | 0.772 | NA | NA |  |
| Oenanthe | Oenanthe_lugens | directional migratory | Non-tropical | 4657457 | April | May | June |  | 6.54 | 0.300 | 0.230 | 0.244 |  |
| Oenanthe | Oenanthe_lugentoides | resident | Non-tropical | 352627 | April | May | June |  | 5.53 | 0.266 | NA | NA |  |
| Oenanthe | Oenanthe_lugubris | resident | Tropical | 459468 | April | May | June | Close relative (Oenanthe lugens) | 5.94 | 0.274 | NA | NA |  |
| Oenanthe | Oenanthe_melanura | resident | Non-tropical | 3217034 | April | May | June |  | 6.14 | 0.316 | NA | NA |  |
| Oenanthe | Oenanthe_moesta | resident | Non-tropical | 1294994 | March | April | May |  | 5.59 | 0.540 | NA | NA |  |
| Oenanthe | Oenanthe_monacha | resident | Non-tropical | 1519762 | April | May | June |  | 6.62 | 0.314 | NA | NA |  |
| Oenanthe | Oenanthe_monticola | resident | Tropical | 1856762 | September | October | November |  | 6.05 | 0.670 | NA | NA |  |
| Oenanthe | Oenanthe_oenanthe | directional migratory | Tropical | 42940839 | May | June | July |  | 6.94 | 0.418 | 0.226 | 0.198 |  |
| Oenanthe | Oenanthe_phillipsi | resident | Non-tropical | 596237 | April | May | June |  | 4.75 | 0.303 | NA | NA |  |
| Oenanthe | Oenanthe_picata | directional migratory | Non-tropical | 3408303 | April | May | June |  | 6.50 | 0.388 | 0.275 | 0.362 |  |
| Oenanthe | Oenanthe_pileata | dispersive migratory | Tropical | 6441956 | June | August | October |  | NA | NA | NA | NA | Migratory status |
| Oenanthe | Oenanthe_pleschanka | directional migratory | Tropical | 15564389 | May | June | July |  | 6.20 | 0.553 | 0.519 | 0.006 |  |
| Oenanthe | Oenanthe_scotocerca | resident | Non-tropical | 771752 | March | April | May |  | 6.24 | 0.665 | NA | NA |  |
| Oenanthe | Oenanthe_xanthoprymna | directional migratory | Non-tropical | 3082682 | June | July | August |  | 5.85 | 0.070 | 0.024 | 0.000 |  |
| Cardinalidae | Passerina_amoena | directional migratory | Non-tropical | 3868335 | June | July | August |  | 6.99 | 0.193 | 0.004 | 0.006 |  |
| Cardinalidae | Passerina_caerulea | directional migratory | Non-tropical | 7212397 | April | May | June |  | 7.87 | 0.177 | 0.441 | 0.234 |  |
| Cardinalidae | Passerina_ciris | directional migratory | Non-tropical | 3225589 | March | April | May |  | 7.28 | 0.119 | 0.415 | 0.517 |  |
| Cardinalidae | Passerina_cyanea | directional migratory | Non-tropical | 6964797 | May | June | July |  | 7.50 | 0.282 | 0.322 | 0.006 |  |
| Cardinalidae | Passerina_leclancherii | resident | Non-tropical | 204479 | May | June | July |  | 6.46 | 0.012 | NA | NA |  |
| Cardinalidae | Passerina_rositae | resident | Non-tropical | 20281 | June | July | August |  | 5.23 | 0 | NA | NA |  |
| Cardinalidae | Passerina_versicolor | directional migratory | Non-tropical | 1635179 | April | May | June |  | 7.38 | 0.085 | 0.233 | 0.167 |  |
| Oenanthe | Pentholaea_albifrons | resident | Non-tropical | 2703479 | March | April | May |  | 6.59 | 0.340 | NA | NA |  |
| Oenanthe | Pentholaea_arnotti | resident | Tropical | 3420283 | August | October | December |  | 6.49 | 0.626 | NA | NA |  |
| Oenanthe | Pentholaea_collaris | resident | Tropical | 507052 | September | October | November | Subspecies of Myrmecocichla arnotti | 5.87 | 0.072 | NA | NA |  |
| Cardinalidae | Periporphyrus_erythromelas | resident | Tropical | 857650 | February | March | April | Close relative (Caryothraustes_canadensis) | 6.39 | 0.145 | NA | NA |  |
| Hirundinidae | Petrochelidon_ariel | directional migratory | Tropical | 5284479 | October | November | December |  | 7.07 | 0.543 | 0.492 | 0.512 |  |
| Hirundinidae | Petrochelidon_fluvicola | dispersive migratory | Non-tropical | 2923972 | January | April | August |  | NA | NA | NA | NA | Migratory status |
| Hirundinidae | Petrochelidon_fuliginosa | resident | Tropical | 282365 | April | May | June |  | 5.57 | 0.392 | NA | NA |  |
| Hirundinidae | Petrochelidon_fulva | directional migratory | Non-tropical | 1165793 | April | June | August |  | 6.59 | 0.194 | 0.230 | 0.222 |  |
| Hirundinidae | Petrochelidon_nigricans | directional migratory | Tropical | 6819245 | September | October | November |  | 7.19 | 0.450 | 0.558 | 0.608 |  |
| Hirundinidae | Petrochelidon_perdita | unknown | Non-tropical | 3329 | February | March | April |  | NA | NA | NA | NA | Migratory status |
| Hirundinidae | Petrochelidon_preussi | dispersive migratory | Non-tropical | 2846429 | March | April | May |  | NA | NA | NA | NA | Migratory status |
| Hirundinidae | Petrochelidon_pyrrhonota | directional migratory | Tropical | 28024969 | May | June | July |  | 7.16 | 0.396 | 0.650 | 0.242 |  |
| Hirundinidae | Petrochelidon_rufigula | directional migratory | Tropical | 1618892 | July | August | September |  | 6.22 | 0.071 | 0.076 | 0.068 |  |
| Hirundinidae | Petrochelidon_rufocollaris | resident | Tropical | 226317 | April | May | June |  | 5.38 | 0.555 | NA | NA |  |
| Hirundinidae | Petrochelidon_spilodera | directional migratory | Tropical | 1552061 | November | January | February |  | 6.15 | 0.116 | 0.159 | 0.019 |  |
| Hirundinidae | Phedina_borbonica | dispersive migratory | Tropical | 687474 | August | September | October |  | NA | NA | NA | NA | Migratory status |
| Hirundinidae | Phedina_brazzae | resident | Tropical | 220480 | July | August | September |  | 5.70 | 0.034 | NA | NA |  |
| Cardinalidae | Pheucticus_aureoventris | dispersive migratory | Tropical | 1580972 | November | December | January |  | NA | NA | NA | NA | Migratory status |
| Cardinalidae | Pheucticus_chrysogaster | resident | Tropical | 597859 | March | April | May |  | 7.13 | 0.689 | NA | NA |  |
| Cardinalidae | Pheucticus_chrysopeplus | directional migratory | Non-tropical | 480571 | June | July | August |  | 7.24 | 0.023 | 0.023 | 0.013 |  |
| Cardinalidae | Pheucticus_ludovicianus | directional migratory | Tropical | 7024934 | June | July | August |  | 6.70 | 0.442 | 0.204 | 0.000 |  |
| Cardinalidae | Pheucticus_melanocephalus | directional migratory | Non-tropical | 5338176 | April | May | June |  | 7.48 | 0.168 | 0.232 | 0.348 |  |
| Cardinalidae | Pheucticus_tibialis | resident | Non-tropical | 72474 | March | April | May |  | 6.25 | 0.075 | NA | NA |  |
| Oenanthe | Pinarochroa_sordida | resident | Tropical | 413577 | February | March | May |  | 6.17 | 0.405 | NA | NA |  |
| Cardinalidae | Piranga_bidentata | resident | Non-tropical | 1063149 | April | May | June |  | 7.43 | 0.273 | NA | NA |  |
| Cardinalidae | Piranga_erythrocephala | resident | Non-tropical | 443127 | May | June | July | Close relative (Piranga_hepatica) | 7.34 | 0.020 | NA | NA |  |
| Cardinalidae | Piranga_flava | directional migratory | Tropical | 5670146 | October | November | December |  | 7.53 | 0.416 | 0.403 | 0.397 |  |
| Cardinalidae | Piranga_hepatica | directional migratory | Non-tropical | 1904246 | May | June | July |  | 7.52 | 0.113 | 0.151 | 0.097 |  |
| Cardinalidae | Piranga_leucoptera | resident | Tropical | 1646860 | May | June | July | Close relative (Piranga_hepatica) | 7.03 | 0.327 | NA | NA |  |
| Cardinalidae | Piranga_ludoviciana | directional migratory | Non-tropical | 5199162 | May | June | July |  | 7.56 | 0.178 | 0.196 | 0.047 |  |
| Cardinalidae | Piranga_lutea | resident | Tropical | 1172864 | July | September | October |  | 7.05 | 0.368 | NA | NA |  |
| Cardinalidae | Piranga_olivacea | directional migratory | Tropical | 5911574 | May | June | July |  | 7.03 | 0.150 | 0.486 | 0.009 |  |
| Cardinalidae | Piranga_roseogularis | resident | Non-tropical | 165832 | May | June | July | Close relative (Piranga_hepatica) | 5.98 | 0 | NA | NA |  |
| Cardinalidae | Piranga_rubra | directional migratory | Tropical | 9173932 | May | June | July |  | 7.76 | 0.180 | 0.405 | 0.004 |  |
| Cardinalidae | Piranga_rubriceps | resident | Tropical | 355687 | September | October | November |  | 5.99 | 0.691 | NA | NA |  |
| Xolmiini | Polioxolmis_rufipennis | resident | Tropical | 627257 | December | January | February |  | 7.38 | 0.183 | NA | NA |  |
| Hirundinidae | Progne_chalybea | directional migratory | Tropical | 15258580 | April | May | June |  | 6.82 | 0.673 | 0.663 | 0.680 |  |
| Hirundinidae | Progne_cryptoleuca | directional migratory | Non-tropical | 110178 | May | June | July |  | NA | NA | NA | NA | Small range |
| Hirundinidae | Progne_dominicensis | directional migratory | Non-tropical | 106756 | April | May | June |  | NA | NA | NA | NA | Small range |
| Hirundinidae | Progne_elegans | directional migratory | Tropical | 4868050 | November | December | January |  | 6.36 | 0.029 | 0.351 | 0.047 |  |
| Hirundinidae | Progne_modesta | resident | Tropical | 7236 | February | March | April |  | NA | NA | NA | NA | Small range |
| Hirundinidae | Progne_murphyi | resident | Tropical | 120128 | February | March | April |  | 5.19 | 0.517 | NA | NA |  |
| Hirundinidae | Progne_sinaloae | directional migratory | Non-tropical | 400589 | April | May | June |  | NA | NA | NA | NA | Small range |
| Hirundinidae | Progne_subis | directional migratory | Tropical | 18939902 | April | May | June |  | 7.05 | 0.379 | 0.658 | 0.337 |  |
| Hirundinidae | Progne_tapera | directional migratory | Tropical | 15215419 | December | January | February |  | 7.15 | 0.590 | 0.630 | 0.546 |  |
| Hirundinidae | Psalidoprocne_albiceps | directional migratory | Tropical | 1323761 | February | June | October |  | 6.18 | 0.721 | 0.727 | 0.749 |  |
| Hirundinidae | Psalidoprocne_fuliginosa | resident | Non-tropical | 31827 | December | January | February |  | 4.65 | 0 | NA | NA |  |
| Hirundinidae | Psalidoprocne_nitens | resident | Tropical | 2241219 | February | May | August |  | 6.35 | 0.888 | NA | NA |  |
| Hirundinidae | Psalidoprocne_obscura | dispersive migratory | Non-tropical | 1561613 | June | July | August |  | NA | NA | NA | NA | Migratory status |
| Hirundinidae | Psalidoprocne_pristoptera | directional migratory | Tropical | 8884728 | April | June | August |  | 6.79 | 0.730 | 0.730 | 0.730 |  |
| Hirundinidae | Pseudhirundo_griseopyga | dispersive migratory | Tropical | 6557321 | June | July | August |  | NA | NA | NA | NA | Migratory status |
| Hirundinidae | Pseudochelidon_eurystomina | directional migratory | Tropical | 541870 | February | March | April |  | 5.50 | 0.064 | 0.160 | 0.326 |  |
| Hirundinidae | Pseudochelidon_sirintarae | directional migratory | Non-tropical | 23745 | February | March | April |  | NA | NA | NA | NA | Small range |
| Turdus | Psophocichla_litsitsirupa | resident | Tropical | 4621273 | September | October | November |  | 6.34 | 0.626 | NA | NA |  |
| Hirundinidae | Ptyonoprogne_concolor | resident | Non-tropical | 3474295 | February | July | August |  | 7.22 | 0.775 | NA | NA |  |
| Hirundinidae | Ptyonoprogne_fuligula | dispersive migratory | Tropical | 7244781 | February | June | October |  | NA | NA | NA | NA | Migratory status |
| Hirundinidae | Ptyonoprogne_obsoleta | resident | Non-tropical | 6159945 | February | March | April |  | 6.57 | 0.433 | NA | NA |  |
| Hirundinidae | Ptyonoprogne_rupestris | directional migratory | Non-tropical | 12590933 | May | June | July |  | 7.37 | 0.302 | 0.073 | 0.079 |  |
| Cardinalidae | Rhodothraupis_celaeno | resident | Non-tropical | 168462 | April | May | June |  | 6.27 | 0.136 | NA | NA |  |
| Hirundinidae | Riparia_chinensis | dispersive migratory | Non-tropical | 3711768 | March | July | November | Subspecies of Riparia paludicola | NA | NA | NA | NA | Migratory status |
| Hirundinidae | Riparia_cincta | directional migratory | Tropical | 9424303 | March | July | November |  | 6.90 | 0.561 | 0.765 | 0.779 |  |
| Hirundinidae | Riparia_congica | resident | Tropical | 194275 | February | March | April |  | 5.16 | 0.232 | NA | NA |  |
| Hirundinidae | Riparia_diluta | directional migratory | Non-tropical | 12776449 | February | March | April |  | 7.19 | 0.118 | 0.204 | 0.569 |  |
| Hirundinidae | Riparia_paludicola | dispersive migratory | Tropical | 7762717 | March | July | November |  | NA | NA | NA | NA | Migratory status |
| Hirundinidae | Riparia_riparia | directional migratory | Tropical | 72465529 | May | June | July |  | 7.25 | 0.400 | 0.576 | 0.159 |  |
| Xolmiini | Satrapa_icterophrys | directional migratory | Tropical | 8290623 | October | December | January |  | 7.96 | 0.551 | 0.566 | 0.529 |  |
| Oenanthe | Saxicola_caprata | directional migratory | Tropical | 9713647 | March | April | June |  | 7.29 | 0.465 | 0.486 | 0.534 |  |
| Oenanthe | Saxicola_dacotiae | resident | Non-tropical | 2009 | February | March | April |  | NA | NA | NA | NA | Small range |
| Oenanthe | Saxicola_ferreus | directional migratory | Non-tropical | 5861028 | April | May | June |  | 7.08 | 0.430 | 0.306 | 0.342 |  |
| Oenanthe | Saxicola_gutturalis | resident | Tropical | 15689 | October | November | December |  | NA | NA | NA | NA | Small range |
| Oenanthe | Saxicola_insignis | directional migratory | Non-tropical | 881490 | June | July | August |  | 5.47 | 0.143 | 0.003 | 0.000 |  |
| Oenanthe | Saxicola_jerdoni | resident | Non-tropical | 563846 | March | April | May |  | 6.34 | 0.109 | NA | NA |  |
| Oenanthe | Saxicola_leucurus | resident | Non-tropical | 809849 | March | April | May |  | 6.45 | 0.464 | NA | NA |  |
| Oenanthe | Saxicola_macrorhynchus | directional migratory | Non-tropical | 716491 | April | May | June |  | 6.18 | 0.190 | 0.190 | 0.190 |  |
| Oenanthe | Saxicola_rubetra | directional migratory | Tropical | 17739735 | May | June | July |  | 6.40 | 0.463 | 0.330 | 0.029 |  |
| Oenanthe | Saxicola_rubicola | directional migratory | NA | NA | April | May | June |  | NA | NA | NA | NA | No data |
| Oenanthe | Saxicola_sibilla | resident | Tropical | 472840 | August | September | October |  | 6.08 | 0.277 | NA | NA |  |
| Oenanthe | Saxicola_stejnegeri | directional migratory | NA | NA | May | June | July |  | NA | NA | NA | NA | No data |
| Oenanthe | Saxicola_tectes | resident | Tropical | 3038 | November | December | January |  | NA | NA | NA | NA | Small range |
| Oenanthe | Saxicola_torquatus | resident | Tropical | 48003261 | May | July | September |  | 7.22 | 0.682 | NA | NA |  |
| Setophaga | Setophaga_adelaidae | resident | Non-tropical | 8892 | April | May | June |  | NA | NA | NA | NA | Small range |
| Setophaga | Setophaga_aestiva | directional migratory | NA | NA | May | June | July |  | NA | NA | NA | NA | No data |
| Setophaga | Setophaga_americana | directional migratory | Non-tropical | 4594806 | May | June | July |  | 6.22 | 0.456 | 0.231 | 0.039 |  |
| Setophaga | Setophaga_angelae | resident | Non-tropical | 8892 | April | May | June |  | NA | NA | NA | NA | Small range |
| Setophaga | Setophaga_auduboni | directional migratory | NA | NA | May | June | July |  | NA | NA | NA | NA | No data |
| Setophaga | Setophaga_caerulescens | directional migratory | Non-tropical | 1924937 | May | June | July |  | 6.04 | 0.130 | 0.428 | 0.003 |  |
| Setophaga | Setophaga_castanea | directional migratory | Non-tropical | 3920861 | May | June | July |  | 6.18 | 0.186 | 0.377 | 0.000 |  |
| Setophaga | Setophaga_cerulea | directional migratory | Tropical | 3199168 | May | June | July |  | 6.70 | 0.065 | 0.429 | 0.000 |  |
| Setophaga | Setophaga_chrysoparia | directional migratory | Non-tropical | 306461 | April | May | June |  | 6.22 | 0.046 | 0.280 | 0.063 |  |
| Setophaga | Setophaga_citrina | directional migratory | Non-tropical | 3101941 | June | July | August |  | 5.98 | 0.337 | 0.177 | 0.002 |  |
| Setophaga | Setophaga_coronata | directional migratory | Non-tropical | 16886961 | May | June | July |  | 7.59 | 0.467 | 0.200 | 0.177 |  |
| Setophaga | Setophaga_delicata | resident | Non-tropical | 2113 | April | May | June | Close relative (Setophaga adelaidae) | NA | NA | NA | NA | Small range |
| Setophaga | Setophaga_discolor | directional migratory | Non-tropical | 2429167 | May | June | July |  | 5.93 | 0.087 | 0.182 | 0.000 |  |
| Setophaga | Setophaga_dominica | directional migratory | Non-tropical | 3529783 | May | June | July |  | 6.41 | 0.211 | 0.344 | 0.000 |  |
| Setophaga | Setophaga_flavescens | resident | Non-tropical | 2759 | May | June | July |  | NA | NA | NA | NA | Small range |
| Setophaga | Setophaga_fusca | directional migratory | Tropical | 4765330 | May | June | July |  | 6.78 | 0.205 | 0.536 | 0.003 |  |
| Setophaga | Setophaga_goldmani | resident | NA | NA | May | June | July |  | NA | NA | NA | NA | No data |
| Setophaga | Setophaga_graciae | directional migratory | Non-tropical | 1617373 | May | June | July |  | 7.19 | 0.113 | 0.164 | 0.110 |  |
| Setophaga | Setophaga_kirtlandii | directional migratory | Non-tropical | 33300 | May | June | July |  | NA | NA | NA | NA | Small range |
| Setophaga | Setophaga_magnolia | directional migratory | Non-tropical | 5796050 | June | July | August |  | 5.83 | 0.369 | 0.181 | 0.000 |  |
| Setophaga | Setophaga_nigrescens | directional migratory | Non-tropical | 2686887 | May | June | July |  | 6.61 | 0.052 | 0.015 | 0.044 |  |
| Setophaga | Setophaga_occidentalis | directional migratory | Non-tropical | 1309232 | May | June | July |  | 6.70 | 0.254 | 0.209 | 0.003 |  |
| Setophaga | Setophaga_palmarum | directional migratory | Non-tropical | 4918077 | May | June | July |  | 6.46 | 0.215 | 0.366 | 0.000 |  |
| Setophaga | Setophaga_pensylvanica | directional migratory | Non-tropical | 4643592 | May | June | July |  | 6.23 | 0.225 | 0.244 | 0.005 |  |
| Setophaga | Setophaga_petechia | resident | Tropical | 19244540 | April | May | June |  | 7.71 | 0.199 | NA | NA |  |
| Setophaga | Setophaga_pharetra | resident | Non-tropical | 11021 | March | April | May |  | NA | NA | NA | NA | Small range |
| Setophaga | Setophaga_pinus | directional migratory | Non-tropical | 3039324 | April | May | June |  | 6.30 | 0.223 | 0.130 | 0.209 |  |
| Setophaga | Setophaga_pitiayumi | resident | Tropical | 9730576 | March | June | August |  | 7.43 | 0.706 | NA | NA |  |
| Setophaga | Setophaga_pityophila | resident | Non-tropical | 21666 | March | April | May |  | 5.54 | 0.044 | NA | NA |  |
| Setophaga | Setophaga_plumbea | resident | Non-tropical | 2437 | April | May | June |  | NA | NA | NA | NA | Small range |
| Setophaga | Setophaga_ruticilla | directional migratory | Tropical | 12309319 | May | June | July |  | 7.03 | 0.408 | 0.319 | 0.078 |  |
| Setophaga | Setophaga_striata | directional migratory | Tropical | 11518441 | May | June | July |  | 7.11 | 0.197 | 0.561 | 0.023 |  |
| Setophaga | Setophaga_subita | resident | Non-tropical | 436 | April | May | June | Close relative (Setophaga adelaidae) | NA | NA | NA | NA | Small range |
| Setophaga | Setophaga_tigrina | directional migratory | Non-tropical | 4149085 | June | July | August |  | 5.54 | 0.572 | 0.109 | 0.000 |  |
| Setophaga | Setophaga_townsendi | directional migratory | Non-tropical | 4130350 | May | June | July |  | 7.04 | 0.240 | 0.151 | 0.021 |  |
| Setophaga | Setophaga_virens | directional migratory | Non-tropical | 5434409 | May | June | July |  | 6.38 | 0.422 | 0.198 | 0.052 |  |
| Setophaga | Setophaga_vitellina | resident | Non-tropical | 281 | April | May | June |  | NA | NA | NA | NA | Small range |
| Cardinalidae | Spiza_americana | directional migratory | Non-tropical | 5598238 | May | June | July |  | 6.97 | 0.293 | 0.238 | 0.001 |  |
| Hirundinidae | Stelgidopteryx_ruficollis | directional migratory | Tropical | 13898140 | April | May | June |  | 6.82 | 0.726 | 0.748 | 0.720 |  |
| Hirundinidae | Stelgidopteryx_serripennis | directional migratory | Non-tropical | 10737874 | March | April | May |  | 7.20 | 0.272 | 0.440 | 0.634 |  |
| Hirundinidae | Tachycineta_albilinea | resident | Non-tropical | 1106031 | March | April | May |  | 6.34 | 0.325 | NA | NA |  |
| Hirundinidae | Tachycineta_albiventer | directional migratory | Tropical | 12436158 | February | March | April |  | 6.78 | 0.623 | 0.607 | 0.633 |  |
| Hirundinidae | Tachycineta_bicolor | directional migratory | Non-tropical | 15549291 | May | June | July |  | 6.69 | 0.460 | 0.215 | 0.093 |  |
| Hirundinidae | Tachycineta_cyaneoviridis | dispersive migratory | Non-tropical | 12781 | May | June | July |  | NA | NA | NA | NA | Migratory status |
| Hirundinidae | Tachycineta_euchrysea | resident | Non-tropical | 44989 | June | July | August |  | 4.63 | 0 | NA | NA |  |
| Hirundinidae | Tachycineta_leucopyga | directional migratory | Tropical | 4559783 | October | November | December |  | 6.89 | 0.331 | 0.325 | 0.124 |  |
| Hirundinidae | Tachycineta_leucorrhoa | directional migratory | Tropical | 6890623 | October | November | December |  | 6.50 | 0.288 | 0.240 | 0.195 |  |
| Hirundinidae | Tachycineta_stolzmanni | resident | Non-tropical | 92643 | January | February | March |  | 5.21 | 0.423 | NA | NA |  |
| Hirundinidae | Tachycineta_thalassina | directional migratory | Non-tropical | 7547618 | May | June | July |  | 6.79 | 0.123 | 0.073 | 0.197 |  |
| Oenanthe | Thamnolaea_cinnamomeiventris | resident | Tropical | 3677478 | November | March | July |  | 6.49 | 0.769 | NA | NA |  |
| Oenanthe | Thamnolaea_coronata | resident | Non-tropical | 863167 | May | June | July |  | 6.33 | 0.091 | NA | NA |  |
| Turdus | Turdus_abyssinicus | resident | Tropical | 1446670 | March | June | August |  | 6.43 | 0.695 | NA | NA |  |
| Turdus | Turdus_albicollis | resident | Tropical | 8584114 | October | November | December |  | 6.61 | 0.584 | NA | NA |  |
| Turdus | Turdus_albocinctus | dispersive migratory | Non-tropical | 640411 | April | May | June |  | NA | NA | NA | NA | Migratory status |
| Turdus | Turdus_amaurochalinus | directional migratory | Tropical | 8756466 | February | October | December |  | 6.89 | 0.367 | 0.371 | 0.209 |  |
| Turdus | Turdus_assimilis | resident | Non-tropical | 984561 | April | May | June |  | 6.54 | 0.405 | NA | NA |  |
| Turdus | Turdus_atrogularis | directional migratory | Non-tropical | 8408414 | May | June | July |  | 6.65 | 0.342 | 0.057 | 0.000 |  |
| Turdus | Turdus_aurantius | resident | Non-tropical | 11021 | May | June | July |  | NA | NA | NA | NA | Small range |
| Turdus | Turdus_bewsheri | resident | Tropical | 1671 | August | September | October |  | NA | NA | NA | NA | Small range |
| Turdus | Turdus_boulboul | directional migratory | Non-tropical | 1448808 | April | May | June |  | 6.93 | 0.356 | 0.103 | 0.330 |  |
| Turdus | Turdus_cardis | directional migratory | Non-tropical | 1107354 | May | June | July |  | 5.83 | 0.290 | 0.009 | 0.026 |  |
| Turdus | Turdus_celaenops | resident | Non-tropical | 1387 | April | May | June |  | NA | NA | NA | NA | Small range |
| Turdus | Turdus_chiguanco | resident | Tropical | 1315594 | April | May | June |  | 6.26 | 0.467 | NA | NA |  |
| Turdus | Turdus_chrysolaus | directional migratory | Non-tropical | 1290933 | June | July | August |  | 5.67 | 0.204 | 0.072 | 0.000 |  |
| Turdus | Turdus_daguae | resident | Tropical | 198903 | April | May | June |  | 5.29 | 0.624 | NA | NA |  |
| Turdus | Turdus_dissimilis | dispersive migratory | Non-tropical | 1377645 | April | May | June |  | NA | NA | NA | NA | Migratory status |
| Turdus | Turdus_eunomus | directional migratory | Non-tropical | 7257360 | June | July | August |  | 6.34 | 0.386 | 0.021 | 0.000 |  |
| Turdus | Turdus_falcklandii | resident | Non-tropical | 756465 | October | November | December |  | 6.53 | 0.065 | NA | NA |  |
| Turdus | Turdus_feae | directional migratory | Non-tropical | 356170 | May | June | July |  | 6.00 | 0.025 | 0.022 | 0.000 |  |
| Turdus | Turdus_flavipes | directional migratory | Tropical | 1153499 | November | December | January |  | 6.06 | 0.284 | 0.291 | 0.272 |  |
| Turdus | Turdus_fulviventris | resident | Tropical | 220011 | May | June | July |  | 5.50 | 0.319 | NA | NA |  |
| Turdus | Turdus_fumigatus | resident | Tropical | 4514381 | February | June | December |  | 6.36 | 0.856 | NA | NA |  |
| Turdus | Turdus_fuscater | resident | Tropical | 836001 | March | April | June |  | 6.23 | 0.614 | NA | NA |  |
| Turdus | Turdus_grayi | resident | Non-tropical | 1111860 | April | May | June |  | 6.10 | 0.255 | NA | NA |  |
| Turdus | Turdus_haplochrous | resident | Tropical | 119219 | April | May | June | Close relative (Turdus chiguanco) | 5.21 | 0.051 | NA | NA |  |
| Turdus | Turdus_hauxwelli | resident | Tropical | 3142036 | February | March | April |  | 6.14 | 0.419 | NA | NA |  |
| Turdus | Turdus_helleri | resident | Non-tropical | 12287 | March | April | May |  | NA | NA | NA | NA | Small range |
| Turdus | Turdus_hortulorum | directional migratory | Non-tropical | 3152073 | May | June | July |  | 5.79 | 0.086 | 0.009 | 0.000 |  |
| Turdus | Turdus_ignobilis | resident | Tropical | 4077041 | February | April | June |  | 6.48 | 0.726 | NA | NA |  |
| Turdus | Turdus_iliacus | directional migratory | Non-tropical | 18013755 | April | May | June |  | 6.74 | 0.366 | 0.513 | 0.154 |  |
| Turdus | Turdus_infuscatus | resident | Non-tropical | 265641 | May | June | July |  | 6.02 | 0.112 | NA | NA |  |
| Turdus | Turdus_jamaicensis | resident | Non-tropical | 11021 | April | May | June |  | NA | NA | NA | NA | Small range |
| Turdus | Turdus_kessleri | dispersive migratory | Non-tropical | 1701708 | May | June | July |  | NA | NA | NA | NA | Migratory status |
| Turdus | Turdus_lawrencii | resident | Tropical | 3103716 | December | January | February |  | 6.19 | 0.504 | NA | NA |  |
| Turdus | Turdus_leucomelas | resident | Tropical | 7646988 | November | December | January |  | 6.68 | 0.552 | NA | NA |  |
| Turdus | Turdus_leucops | resident | Tropical | 818871 | February | March | April |  | 6.09 | 0.561 | NA | NA |  |
| Turdus | Turdus_lherminieri | resident | Non-tropical | 3998 | April | May | June |  | NA | NA | NA | NA | Small range |
| Turdus | Turdus_libonyana | resident | Tropical | 4824453 | September | October | November |  | 6.32 | 0.418 | NA | NA |  |
| Turdus | Turdus_ludoviciae | resident | Non-tropical | 59710 | May | June | July |  | 4.80 | 0.003 | NA | NA |  |
| Turdus | Turdus_maculirostris | resident | Tropical | 171415 | January | February | March |  | 5.40 | 0.271 | NA | NA |  |
| Turdus | Turdus_maranonicus | resident | Non-tropical | 97899 | January | February | March | Close relative (Turdus maculirostris) | 5.39 | 0.077 | NA | NA |  |
| Turdus | Turdus_maximus | resident | Non-tropical | 572862 | May | June | July |  | 6.69 | 0.240 | NA | NA |  |
| Turdus | Turdus_menachensis | resident | Non-tropical | 116014 | April | May | June |  | 5.75 | 0.225 | NA | NA |  |
| Turdus | Turdus_merula | directional migratory | Non-tropical | 17432667 | April | June | July |  | 7.33 | 0.356 | 0.318 | 0.277 |  |
| Turdus | Turdus_migratorius | directional migratory | Non-tropical | 18466880 | May | June | July |  | 7.38 | 0.306 | 0.139 | 0.286 |  |
| Turdus | Turdus_mupinensis | resident | Non-tropical | 1839448 | May | June | July |  | 6.79 | 0.018 | NA | NA |  |
| Turdus | Turdus_naumanni | directional migratory | Non-tropical | 4290887 | May | June | July |  | 6.24 | 0.025 | 0.004 | 0.000 |  |
| Turdus | Turdus_nigrescens | resident | Non-tropical | 48063 | March | April | May |  | 5.65 | 0.036 | NA | NA |  |
| Turdus | Turdus_nigriceps | directional migratory | Tropical | 971453 | November | December | January |  | 6.22 | 0.283 | 0.137 | 0.167 |  |
| Turdus | Turdus_nudigenis | resident | Tropical | 1674351 | June | July | August |  | 6.27 | 0.399 | NA | NA |  |
| Turdus | Turdus_obscurus | directional migratory | Tropical | 9818323 | May | June | July |  | 6.54 | 0.331 | 0.516 | 0.000 |  |
| Turdus | Turdus_obsoletus | resident | Tropical | 346795 | April | May | June |  | 5.56 | 0.624 | NA | NA |  |
| Turdus | Turdus_olivaceofuscus | resident | Non-tropical | 839 | October | November | December |  | NA | NA | NA | NA | Small range |
| Turdus | Turdus_olivaceus | resident | Tropical | 496844 | March | July | November |  | 5.79 | 0.714 | NA | NA |  |
| Turdus | Turdus_olivater | resident | Non-tropical | 472109 | March | April | June |  | 5.99 | 0.253 | NA | NA |  |
| Turdus | Turdus_pallidus | directional migratory | Non-tropical | 4966446 | May | June | July |  | 6.54 | 0.197 | 0.135 | 0.001 |  |
| Turdus | Turdus_pelios | resident | Tropical | 8947192 | March | July | November |  | 7.22 | 0.620 | NA | NA |  |
| Turdus | Turdus_philomelos | directional migratory | Non-tropical | 18663870 | May | June | July |  | 6.74 | 0.299 | 0.109 | 0.052 |  |
| Turdus | Turdus_pilaris | directional migratory | Non-tropical | 19541587 | May | June | July |  | 6.94 | 0.226 | 0.128 | 0.026 |  |
| Turdus | Turdus_plebejus | resident | Non-tropical | 243944 | March | April | May |  | 6.32 | 0.208 | NA | NA |  |
| Turdus | Turdus_plumbeus | resident | Non-tropical | 202446 | April | May | June |  | 5.41 | 0.170 | NA | NA |  |
| Turdus | Turdus_poliocephalus | resident | Tropical | 1071887 | April | May | June |  | 6.02 | 0.743 | NA | NA |  |
| Turdus | Turdus_ravidus | resident | Non-tropical | 211 | - | - | - |  | NA | NA | NA | NA | Extinct |
| Turdus | Turdus_reevei | resident | Tropical | 111462 | January | February | March |  | 5.64 | 0.195 | NA | NA |  |
| Turdus | Turdus_roehli | resident | Non-tropical | 48296 | July | August | September |  | 4.58 | 0.050 | NA | NA |  |
| Turdus | Turdus_rubrocanus | directional migratory | Non-tropical | 2025778 | May | June | July |  | 6.45 | 0.116 | 0.048 | 0.082 |  |
| Turdus | Turdus_ruficollis | directional migratory | Non-tropical | 2841132 | May | June | July |  | 5.91 | 0.129 | 0.171 | 0.000 |  |
| Turdus | Turdus_rufitorques | resident | Non-tropical | 161246 | March | April | May | Close relative (Turdus plebejus) | 6.01 | 0.068 | NA | NA |  |
| Turdus | Turdus_rufiventris | resident | Tropical | 5128756 | September | October | November |  | 6.38 | 0.438 | NA | NA |  |
| Turdus | Turdus_rufopalliatus | resident | Non-tropical | 491660 | June | July | August |  | 6.12 | 0.001 | NA | NA |  |
| Turdus | Turdus_sanchezorum | resident | Tropical | 686252 | December | January | February |  | 5.62 | 0.393 | NA | NA |  |
| Turdus | Turdus_serranus | resident | Tropical | 1108691 | May | June | July |  | 6.54 | 0.417 | NA | NA |  |
| Turdus | Turdus_simillimus | directional migratory | Non-tropical | 1455883 | April | July | September |  | 7.17 | 0.181 | 0.222 | 0.190 |  |
| Turdus | Turdus_smithi | resident | Non-tropical | 900040 | September | October | November |  | 5.69 | 0.521 | NA | NA |  |
| Turdus | Turdus_subalaris | directional migratory | Tropical | 2477436 | November | December | January |  | 5.75 | 0.082 | 0.021 | 0.036 |  |
| Turdus | Turdus_swalesi | resident | Non-tropical | 27259 | May | June | July |  | 4.79 | 0 | NA | NA |  |
| Turdus | Turdus_tephronotus | resident | Tropical | 763404 | March | April | May |  | 5.78 | 0.526 | NA | NA |  |
| Turdus | Turdus_torquatus | directional migratory | Non-tropical | 4927859 | May | June | July |  | 6.57 | 0.349 | 0.018 | 0.059 |  |
| Turdus | Turdus_unicolor | directional migratory | Non-tropical | 1656266 | May | June | July |  | 6.59 | 0.406 | 0.015 | 0.187 |  |
| Turdus | Turdus_viscivorus | directional migratory | Non-tropical | 16640278 | April | May | June |  | 6.96 | 0.447 | 0.331 | 0.274 |  |
| Turdus | Turdus_xanthorhynchus | resident | Non-tropical | 177 | October | November | December |  | NA | NA | NA | NA | Small range |
| Vireonidae | Vireo_altiloquus | directional migratory | Tropical | 5214907 | May | June | July |  | 6.52 | 0.104 | 0.610 | 0.013 |  |
| Vireonidae | Vireo_approximans | resident | Non-tropical | 28 | April | May | June |  | NA | NA | NA | NA | Small range |
| Vireonidae | Vireo_atricapilla | directional migratory | Non-tropical | 661500 | May | June | July |  | 6.19 | 0.003 | 0.004 | 0.000 |  |
| Vireonidae | Vireo_bairdi | resident | Non-tropical | 856 | May | June | July |  | NA | NA | NA | NA | Small range |
| Vireonidae | Vireo_bellii | directional migratory | Non-tropical | 3444329 | April | May | June |  | 6.92 | 0.117 | 0.151 | 0.086 |  |
| Vireonidae | Vireo_brevipennis | resident | Non-tropical | 272391 | May | June | July |  | 6.45 | 0.010 | NA | NA |  |
| Vireonidae | Vireo_caribaeus | resident | Non-tropical | 28 | April | May | June |  | NA | NA | NA | NA | Small range |
| Vireonidae | Vireo_carmioli | resident | Non-tropical | 36508 | March | April | May |  | 5.99 | 0.106 | NA | NA |  |
| Vireonidae | Vireo_cassinii | directional migratory | Non-tropical | 2893957 | April | May | June |  | 6.84 | 0.504 | 0.152 | 0.170 |  |
| Vireonidae | Vireo_crassirostris | resident | Non-tropical | 34827 | April | May | June |  | NA | NA | NA | NA | Small range |
| Vireonidae | Vireo_flavifrons | directional migratory | Non-tropical | 5258014 | May | June | July |  | 6.38 | 0.136 | 0.241 | 0.019 |  |
| Vireonidae | Vireo_flavoviridis | directional migratory | Tropical | 3761514 | May | June | July |  | 6.88 | 0.545 | 0.396 | 0.245 |  |
| Vireonidae | Vireo_gilvus | directional migratory | Non-tropical | 10115569 | May | June | July |  | 7.22 | 0.254 | 0.068 | 0.124 |  |
| Vireonidae | Vireo_gracilirostris | resident | Tropical | 19 | May | June | July |  | NA | NA | NA | NA | Small range |
| Vireonidae | Vireo_griseus | directional migratory | Non-tropical | 3702124 | April | May | June |  | 6.64 | 0.326 | 0.454 | 0.185 |  |
| Vireonidae | Vireo_gundlachii | resident | Non-tropical | 110178 | April | May | June |  | 5.39 | 0.032 | NA | NA |  |
| Vireonidae | Vireo_huttoni | resident | Non-tropical | 1795337 | February | March | April |  | 7.40 | 0.230 | NA | NA |  |
| Vireonidae | Vireo_hypochryseus | resident | Non-tropical | 471956 | June | July | August |  | 6.47 | 0.000 | NA | NA |  |
| Vireonidae | Vireo_latimeri | resident | Non-tropical | 7249 | March | April | May |  | NA | NA | NA | NA | Small range |
| Vireonidae | Vireo_leucophrys | resident | Tropical | 1174927 | April | May | June |  | 6.90 | 0.546 | NA | NA |  |
| Vireonidae | Vireo_magister | resident | Non-tropical | 41000 | May | June | July |  | 6.03 | 0.056 | NA | NA |  |
| Vireonidae | Vireo_masteri | resident | Non-tropical | 24559 | July | August | September |  | 5.07 | 0.001 | NA | NA |  |
| Vireonidae | Vireo_modestus | resident | Non-tropical | 11021 | April | May | June |  | NA | NA | NA | NA | Small range |
| Vireonidae | Vireo_nanus | resident | Non-tropical | 52857 | March | April | May |  | 5.43 | 0.437 | NA | NA |  |
| Vireonidae | Vireo_nelsoni | resident | Non-tropical | 306211 | May | June | July |  | 6.64 | 0.009 | NA | NA |  |
| Vireonidae | Vireo_olivaceus | directional migratory | Tropical | 23896240 | May | June | July |  | 7.27 | 0.389 | 0.687 | 0.286 |  |
| Vireonidae | Vireo_osburni | resident | Non-tropical | 11021 | April | May | June |  | NA | NA | NA | NA | Small range |
| Vireonidae | Vireo_pallens | resident | Non-tropical | 413040 | April | May | June |  | 6.76 | 0.305 | NA | NA |  |
| Vireonidae | Vireo_philadelphicus | directional migratory | Non-tropical | 3933175 | June | July | August |  | 5.71 | 0.303 | 0.182 | 0.000 |  |
| Vireonidae | Vireo_plumbeus | directional migratory | Non-tropical | 2647424 | April | May | June |  | 6.83 | 0.148 | 0.179 | 0.265 |  |
| Vireonidae | Vireo_solitarius | directional migratory | Non-tropical | 6750142 | April | May | June |  | 6.76 | 0.390 | 0.423 | 0.145 |  |
| Vireonidae | Vireo_vicinior | directional migratory | Non-tropical | 994336 | April | May | June |  | 6.34 | 0.075 | 0.064 | 0.219 |  |
| Vireonidae | Vireolanius_eximius | resident | Non-tropical | 198008 | May | June | July |  | 6.41 | 0.345 | NA | NA |  |
| Vireonidae | Vireolanius_leucotis | resident | Tropical | 4569658 | May | June | July | Close relative (Vireolanius eximius) | 7.09 | 0.583 | NA | NA |  |
| Vireonidae | Vireolanius_melitophrys | resident | Non-tropical | 404002 | May | June | July |  | 6.41 | 0.020 | NA | NA |  |
| Vireonidae | Vireolanius_pulchellus | resident | Non-tropical | 593882 | March | April | May |  | 6.62 | 0.228 | NA | NA |  |
| Xolmiini | Xolmis_cinereus | resident | Tropical | 7118802 | November | December | January |  | 7.76 | 0.396 | NA | NA |  |
| Xolmiini | Xolmis_coronatus | directional migratory | Tropical | 2401996 | September | October | November |  | 7.69 | 0.106 | 0.384 | 0.331 |  |
| Xolmiini | Xolmis_irupero | resident | Tropical | 3670642 | September | October | November |  | 7.82 | 0.514 | NA | NA |  |
| Xolmiini | Xolmis_pyrope | directional migratory | Non-tropical | 819837 | October | November | December |  | 7.82 | 0.122 | 0.120 | 0.074 |  |
| Xolmiini | Xolmis_rubetra | directional migratory | Non-tropical | 1892750 | October | December | February |  | 7.93 | 0.071 | 0.100 | 0.029 |  |
| Xolmiini | Xolmis_salinarum | resident | Non-tropical | 74157 | September | October | November | Close relative (Xolmis irupero) | 6.24 | 0 | NA | NA |  |
| Xolmiini | Xolmis_velatus | resident | Tropical | 3690383 | November | December | January | Close relative (Xolmis cinereus) | 7.28 | 0.223 | NA | NA |  |
