# Supplementary material for: A tale of two seasons: The link between seasonal migration and climatic niches in passerine birds
Source: Ecol Evol. 2020 Oct 20;10(21):11983–97. doi: 10.1002/ece3.6729 (PMC7663971; doi:10.1002/ece3.6729)
Supplement: Supplementary file 2 — Appendix S1 [file ECE3-10-11983-s002.docx]

**Appendix 1**

**Supplementary methods and results**

**Range maps**

We combined two datasets as described in the main text and below. The GeoMiB database was created specifically to produce the best possible range map for each species classified as at least partially non-resident (migratory or nomadic) in Eyres et al. (2017), by using the best source for seasonal range maps for each region and combining each species’ seasonal range map across regions. The distribution maps in the GeoMiB database are polygon range maps or atlas data (which were summarized into a 1° latitudinal-longitudinal grid and turned into polygon range maps), depending on the source. Sources were chosen as the best available information on species’ seasonal range maps for a region, so maps were compiled for most species from multiple sources. Each source was either downloaded from existing online databases or species maps were scanned from books, geo-referenced, and digitized into a geodatabase (Table 2). As some sources covered the same regions, we applied a priority algorithm that overlaid the source maps in a specific order and then deleted areas covered by higher-priority sources in the lower-priority source maps to derive a final species map (from highest to lowest priority: book sources, regional databases, global maps; Supplementary Table 2). This ensured that sources with higher priority were used for those regions rather than maps from sources with lower priority, unless no map from sources with higher priority was available, in which case lower-priority sources supplied the map. Therefore, maps for each species were pieced together from the best available sources for each region of the species’ occurrence. The GeoMiB database was compiled in PostgrSQL version 9.1/9.6 with PostGIS version 2.0/2.3.2. As most of the underlying data are copyrighted, the database cannot be made fully publicly accessible.

To generate a global dataset across all species (including the resident ones without seasonal range maps, which are not included in GeoMiB), we combined the GeoMiB data with a global breeding range dataset for all species which was available only in a 1° latitudinal-longitudinal grid (see below). We therefore matched the taxonomic names by merging or splitting range maps if necessary, and resampled the GeoMiB polygons into this 1° grid. A species was counted as occurring in the cell if 5% or more of the cell area was covered by the species range map; this rule did not apply for terrestrial species occurring in cells that had islands covering <5% of the cell surface, where any occurrences were counted. At the same time, marine grid cells were removed following the same rules for each terrestrial species, i.e. species that were not classified as either partially or fully marine in Eyres et al. (2017). During gridding, we assigned a single area type for each species record in each grid cell: non-breeding, breeding, or year-round. To determine the area type in a given grid cell where multiple GeoMiB polygons with different range types overlapped, the cell was classified as year-round unless there was >20 times more breeding range area covering the cell than year-round range area (then it was classified as breeding) or >20 times more non-breeding area than year-round (then it was classified as non-breeding).

We then combined the gridded GeoMiB ranges with the Copenhagen database of breeding ranges (CPH database, see Holt et al. (2013) for a description and detailed references; data extracted 24^th^ June 2014). The CPH database is a comprehensive global compilation for all land and non-pelagic species, which is only available in a 1° latitudinal-longitudinal grid. Maps have been validated by ornithological experts and are a conservative estimate of extent-of-occurrence during the breeding season based on museum specimens, published sight records, and spatial distribution of habitats between documented records (based on over 1600 references). Seasonal assignments in the final combined database were as follows: breeding if the cell existed in CPH database and was classified as breeding in GeoMiB (if a breeding grid cell only existed in GeoMiB, it was removed from the range); non-breeding if the cell did not exist in the CPH database and was classified as non-breeding or year-round in GeoMiB; and year-round if the cell existed in the CPH database and was classified as year-round or non-breeding in the GeoMiB database. For species that did not exist in the GeoMiB database (i.e., all fully resident species), all cells were taken from the CPH dataset and categorized as year-round. All automatic range type assignments were manually checked for obvious inconsistencies, and modified if necessary. For calculation of seasonal climatic niches, breeding and year-round records were assigned each breeding month recorded for the species, and non-breeding and year round records were assigned each non-breeding month recorded for the species.

**Niche metrics and overlap calculation**

Seasonal niche overlap was quantified following the methods of Broennimann et al. (2012). Principal component analysis (PCA) was carried out to incorporate information from all six climatic variables and create a two dimensional space in which niche overlap was measured. As different climatic factors might be important for determining each clade’s distribution, we carried out PCA for each clade individually; PCA was carried out across each entire clade to ensure comparability across species within each clade. In order to measure seasonal niche overlap with occurrence points within the bi-dimensional climatic space characterized by the first two principal components, the entire climatic space available to a clade was gridded into a 100 x 100 cells following Broennimann et al. (2012). The occurrences of each species and the climatic space available to each species (see explanation below and figure 1c) in the season of interest were then converted into densities within this grid. To ensure our metrics were independent of the resolution of the grid, kernel density smoothing was used to generate density surfaces.

To ensure that quantification of niches was comparable across all species and accounted for availability of climatic conditions, each clade-wide PCA was carried out including not only the species occurrences but also the climate available to the clade as a whole across both seasons. Species occurrence densities were subsequently divided by the density surface of available climate to give “climatic occupancy values” (Broennimann et al. 2012). The climate available to a species in a season was defined as the climate across all zoogeographic realms that the species inhabits in that season; the climate available to the clade as a whole was defined as all the zoogeographic realms that any member of the clade inhabits (see figure 1c for an example species). Zoogeographic realms were chosen because their borders represent areas of major turnover in species’ distributions and phylogenetic lineages (Holt et al. 2013), so they approximate common frontiers to dispersal processes. The overlap between breeding and non-breeding niches was then calculated for each species based on the climatic occupancy values using Schoeners D, a measure that varies between 0 (no overlap) and 1 (complete overlap)(Warren et al. 2008).

**Influence of geographic range size on niche overlap**

To determine whether geographic range size influenced seasonal niche overlap, we tested whether range size differed between different categories of movement behaviour and for a relationship between range size and seasonal niche overlap. A linear mixed effects model across all species accounting for clade as a random effect showed that directional migrants had on average significantly larger total geographic range sizes than resident species (log transformed range size for migrants =15.54 ± 0.14, for residents = -1.51 ± 0.14, F=121.91, df = 434.9, p<0.001). We therefore tested whether there was a significant relationship between total range size and seasonal niche overlap. Using a linear mixed effects model accounting for clade as a random effect, geographic range size had a significant positive effect on seasonal niche overlap (slope =0.06 ± 0.006, t=10.06, df=431.86, p<0.001, figure S2), showing that species with larger ranges had greater niche overlap.

**Clade specific patterns in overlap values**

To check for clade-specific effects that we could not detect by fitting clade as a random effect, we also ran linear models in which clade was included as a fixed effect. Firstly we fitted a full model with a three-way interaction between breeding location, migratory status and clade and controlling for range size. As there are not enough species in each of these categories for some clades and we run the risk of overfitting the model we additionally ran a second model without breeding location (as above with an interaction between migratory and clade and controlling for range size).

Results from these models were fairly consistent across the clades. In the first model, we found significant main effects of breeding location, migratory status, clade and range. We found no significant interactions with migratory status or breeding location with clade indicating that although niche overlap varied between clades, overall the effects of breeding location and migratory status are consistent across clades (Table S6, Fig. 3 (b-i)). Four clades showed results consistent with the main analysis. Two clades, *Oenanthe* and *Setophaga*, showed the expected results for species breeding outside of the tropics but did not have sufficient tropical breeding species; two further clades (Xolmiini and *Corvus*) did not show results consistent with the main analysis. For these two clades there was no significant difference between overlap of migratory and resident species that breed outside of the tropics (Fig. 3). Despite these slight differences between clades, the simpler model without breeding location showed no interaction between migratory status and clade (Table S7) showing consistently significantly greater overlap in seasonal niches for resident than migratory species in each clade (Fig. S4, Table S7).

**Testing for phylogenetic influence on niche overlap**

To control for phylogenetic relationships within clades more explicitly, in addition to our main analysis in which clade is included as a random effect, we additionally fitted an equivalent model using phylogenetic generalised least-squares regression analyses (PGLS). Seasonal niche overlap was the response variable. Migratory status, breeding location, range size (logged) and the interaction between migratory status and breeding location were included as predictors. PGLS analyses were conducted using the *caper* package in R (Orme et al. 2014). We obtained dated phylogenies from birdtree.org (Jetz et al 2012). PGLS analysis was carried out on a random sample of 100 posterior trees from the full trees built with a Hackett backbone. Results from these 100 runs were summarised by taking the mean. By matching the species available in the phylogenetic datasets to our data on niche quantification, the total number of species was reduced to 415 for these analyses. The results were qualitatively similar when we controlled for the effects of phylogeny below the clade level using PGLS.

**Supplementary figures**


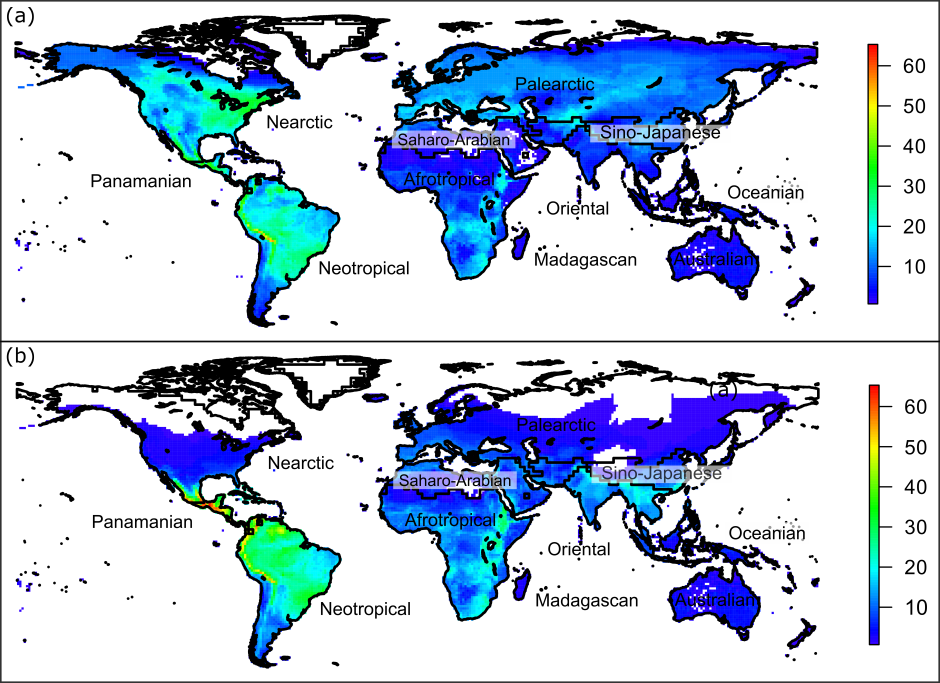


Figure S1. Species richness for the 437 species included in our final analyses. Shown for northern-hemisphere summer (a) and for northern-hemisphere winter (b). Thick lines indicate the 11 terrestrial zoographic realms (Holt et al. 2013). Our clades are distributed widely in both seasons with species present in all 11 zoographic realms.

**
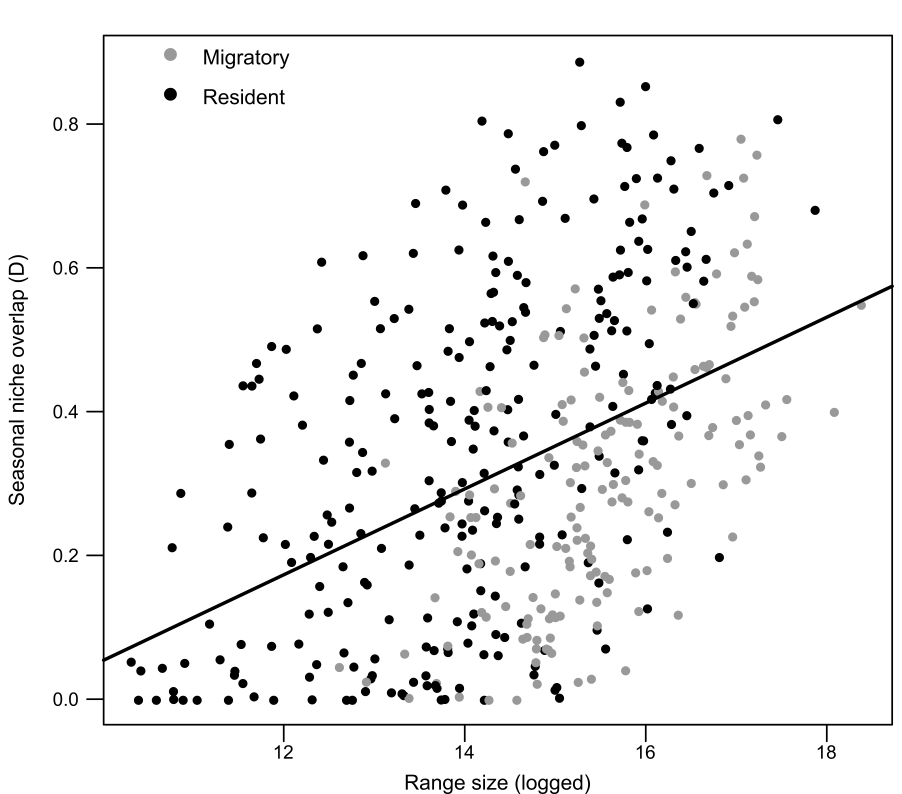
**

**Figure S2.** Relationship between seasonal niche overlap and range size across 8 clades of passerine birds (n = 437). The line shows the results of a simple linear mixed effects model controlling for clade as a random effect. Range size was log-transformed. Niche overlap was calculated from the climatic occupancy values using Schoeners D. Migratory species (grey) had larger average range sizes than resident species (black).


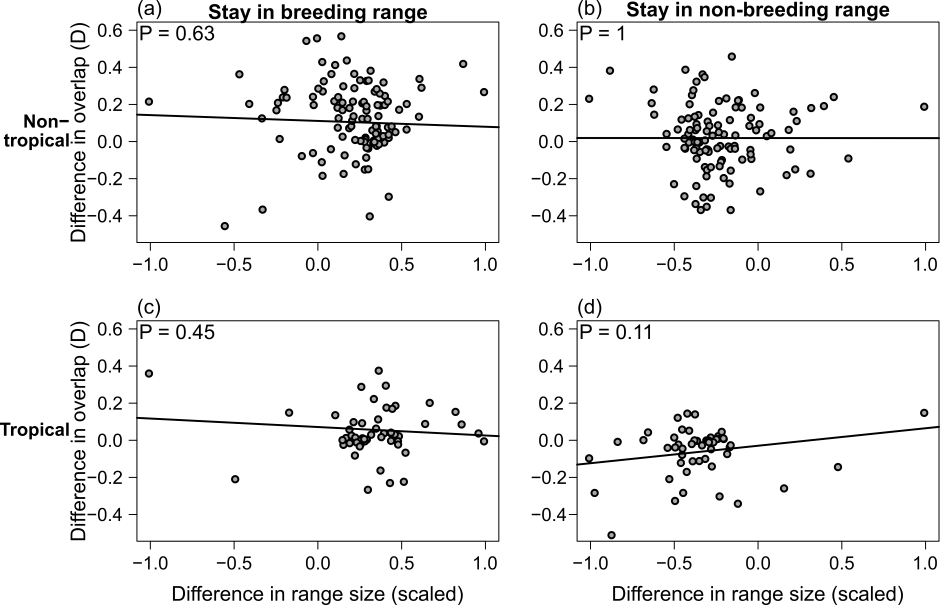


Figure S3. Results of the linear mixed effects model testing for an effect of geographic range size on differences in niche overlap, contrasting the experienced seasonal niche overlap to hypothetical overlap if migratory species stayed year round in either the range they occupy in the breeding season (left, a and c) or the non-breeding season (right, b and d) rather than migrate. Difference in range size among seasons (scaled between -1 and 1) was included as the fixed effect and clade was included as a random effect. This is shown for species breeding outside of the tropics (N=115, top, a and b) and species at least partly breeding in the tropics (N= 53, bottom, c and d). There were no statistically significant relationships between difference in overlap and difference in range size. Only directional migrants were considered (N = 168).


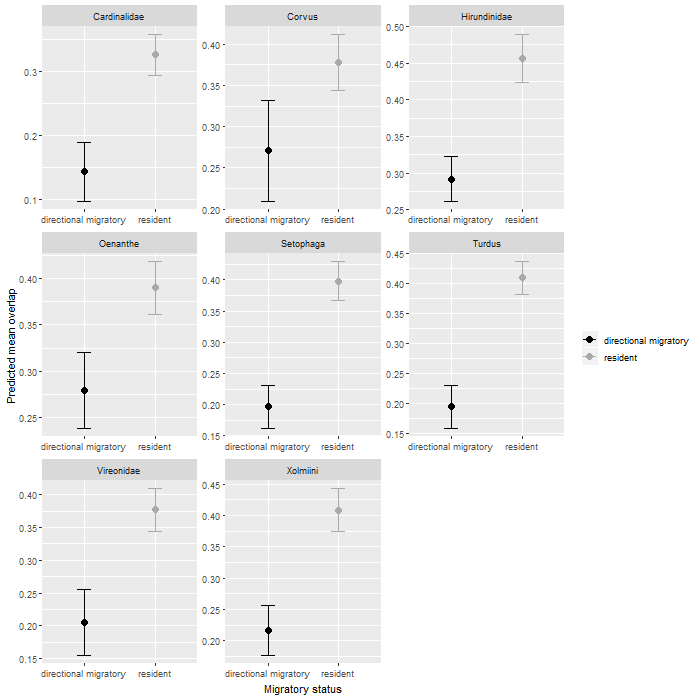


**Figure S4.** Predicted means and standard errors across migratory (M, black) and resident (R, grey) species for seasonal niche overlap for each clade. The linear model (Table S7) included migratory status and clade (and their interaction) as fixed effects. Range size (log-transformed) was also controlled for. Predicted means are shown for the mean range size across all clades. Values of seasonal niche overlap can vary from 0 (no overlap) to 1 (complete overlap).


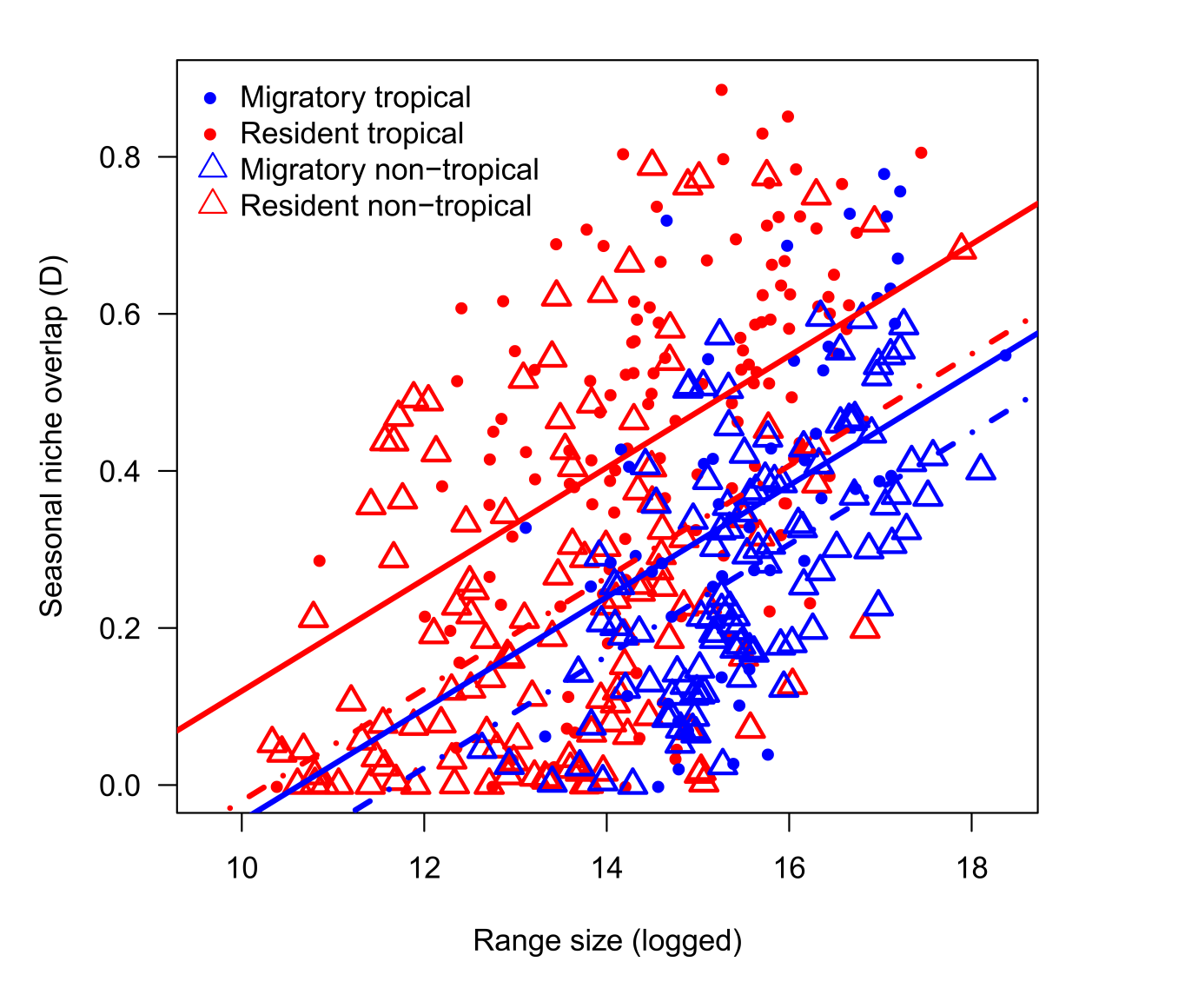


Figure S5. Results of the phylogenetic generalized lease squares analysis (PGLS) (Table S2) testing the effect of migration, breeding location and their interaction on seasonal niche overlap, whilst considering range size and the phylogenetic effect on these traits. Range size measures were log-transformed. Resident species are shown in red and migrants are shown in blue. Continuous lines and filled circles are used for species breeding in the tropics. Dashed lines and empty triangles depict species breeding outside of the tropics.

**Table S1. (Full table in separate file)** Details for all species within our 8 clades. Migratory categorization follows Eyres et al. (2017). Range size is total terrestrial range (sum of breeding plus non-breeding areas). Peak breeding months are shown and source of information denoted. For species included in our study we report climatic niche values. Reason for exclusion from analyses is noted in final column.

| **Clade** | **Species** | **Migratory behaviour** | **Breeding location** | **Range size (Km^2^)** | **Peak breeding months** | | | **Breeding source**  **(unless stated information comes from species description in handbook of birds of the world)** | **Overlap** | **Overlap if**  **stay in**  **winter range** | **Overlap if stay in breeding range** | **Reason for exclusion** |
| --- | --- | --- | --- | --- | --- | --- | --- | --- | --- | --- | --- | --- |
| Xolmiini | *Agriornis_albicauda* | resident | Tropical | 672138 | May | June | July |  | 0.349 | NA | NA |  |
| Xolmiini | *Agriornis_lividus* | resident | Non-tropical | 661396 | October | November | December |  | 0.104 | NA | NA |  |
| Xolmiini | *Agriornis_micropterus* | directional migratory | Tropical | 3197380 | October | November | December |  | 0.268 | 0.304 | 0.330 |  |
| Xolmiini | *Agriornis_montanus* | directional migratory | Tropical | 2178027 | November | December | January |  | 0.360 | 0.340 | 0.344 |  |

**Table S2**: The list of sources used for each region in the GeoMiB database, the source type, and the priority ranking for combining different sources for each species range map.

| **Region** | **Source citation and remarks** | **Source type** | **Priority rank** |
| --- | --- | --- | --- |
| Philippines | Dickinson et al. (2010) | Guidebook | 100 |
| Borneo | Myers (2010) | Guidebook | 90 |
| South Asia | Rasmussen and Anderton (2012) | Guidebook | 80 |
| Australia | Barrett et al. (2003) processed Atlas records for 1998-2012 | Regional database | 70 |
| Europe | Svensson et al. (2010) | Guidebook | 60 |
| West Africa | Borrow and Demey (2001) | Guidebook | 50 |
| Africa | Fry and Urban (1992-2004) as digitized by (Orme et al. 2005) | Regional database | 40 |
| Americas | Ridgely et al. (2012) | Regional database | 30 |
| East Asia | Brazil (2009) | Guidebook | 20 |
| Global | vol. VIII-XV of del Hoyo et al. (2003-2011) | Global database | 10 |

**Table S3.**

Importance of the first six components in the principal components analysis (PCA) for each clade.

| **Clade** |  | **PC1** | **PC2** | **PC3** | **PC4** | **PC5** | **PC6** |
| --- | --- | --- | --- | --- | --- | --- | --- |
| Xolmiini | Standard deviation | 1.863448 | 1.301083 | 0.669921 | 0.559864 | 0.266656 | 0.037393 |
|  | Proportion of Variance | 0.57874 | 0.28214 | 0.0748 | 0.05224 | 0.01185 | 0.00023 |
|  | Cumulative Proportion | 0.57874 | 0.86088 | 0.93567 | 0.98792 | 0.99977 | 1 |
| Hirundinidae | Standard deviation | 1.894609 | 1.351546 | 0.595634 | 0.42229 | 0.221913 | 0.037771 |
|  | Proportion of Variance | 0.59826 | 0.30445 | 0.05913 | 0.02972 | 0.00821 | 0.00024 |
|  | Cumulative Proportion | 0.59826 | 0.9027 | 0.96183 | 0.99155 | 0.99976 | 1 |
| Cardinalidae | Standard deviation | 1.697409 | 1.576645 | 0.615442 | 0.430305 | 0.260842 | 0.032022 |
|  | Proportion of Variance | 0.4802 | 0.4143 | 0.06313 | 0.03086 | 0.01134 | 0.00017 |
|  | Cumulative Proportion | 0.4802 | 0.8945 | 0.95763 | 0.98849 | 0.99983 | 1 |
| Setophaga | Standard deviation | 1.862659 | 1.367443 | 0.629006 | 0.425171 | 0.287518 | 0.038921 |
|  | Proportion of Variance | 0.57825 | 0.31165 | 0.06594 | 0.03013 | 0.01378 | 0.00025 |
|  | Cumulative Proportion | 0.57825 | 0.8899 | 0.95584 | 0.98597 | 0.99975 | 1 |
| Oenanthe | Standard deviation | 1.93568 | 1.288397 | 0.605923 | 0.419259 | 0.220993 | 0.037654 |
|  | Proportion of Variance | 0.62448 | 0.27666 | 0.06119 | 0.0293 | 0.00814 | 0.00024 |
|  | Cumulative Proportion | 0.62448 | 0.90114 | 0.96233 | 0.99162 | 0.99976 | 1 |
| Corvus | Standard deviation | 1.933232 | 1.294084 | 0.603627 | 0.416925 | 0.219792 | 0.038211 |
|  | Proportion of Variance | 0.6229 | 0.27911 | 0.06073 | 0.02897 | 0.00805 | 0.00024 |
|  | Cumulative Proportion | 0.6229 | 0.90201 | 0.96273 | 0.99171 | 0.99976 | 1 |
| Vireonidae | Standard deviation | 1.848555 | 1.386901 | 0.625585 | 0.433709 | 0.279796 | 0.04005 |
|  | Proportion of Variance | 0.56953 | 0.32058 | 0.06523 | 0.03135 | 0.01305 | 0.00027 |
|  | Cumulative Proportion | 0.56953 | 0.89011 | 0.95533 | 0.98668 | 0.99973 | 1 |
| Turdus | Standard deviation | 1.882881 | 1.366952 | 0.598637 | 0.422437 | 0.218935 | 0.03808 |
|  | Proportion of Variance | 0.59087 | 0.31143 | 0.05973 | 0.02974 | 0.00799 | 0.00024 |
|  | Cumulative Proportion | 0.59087 | 0.9023 | 0.96203 | 0.99177 | 0.99976 | 1 |

**Table S4.** Factor loadings by climatic variable for each principal component axis shown for each clade. Climatic variables are: maximum temperature (tmax), minimum temperature (tmin), relative humidity am (rham), relative humidity pm (rhpm), radiation (rad) and precipitation (pre).

| Clade | Climatic variable | PC1 | PC2 | PC3 | PC4 | PC5 | PC6 |
| --- | --- | --- | --- | --- | --- | --- | --- |
| Xolmiini | tmax | 0.315428 | 0.593717 | -0.20911 | -0.27999 | -0.43187 | -0.48926 |
|  | tmin | 0.421116 | 0.452177 | -0.14418 | -0.19877 | 0.472885 | 0.578169 |
|  | rham | 0.482255 | -0.20181 | -0.23676 | 0.496448 | -0.54184 | 0.361381 |
|  | rhpm | 0.501099 | -0.19332 | -0.09239 | 0.360966 | 0.52663 | -0.54347 |
|  | rad | -0.23869 | 0.596371 | 0.347316 | 0.682158 | 0.037358 | -0.00198 |
|  | pre | 0.427226 | -0.09631 | 0.866178 | -0.20001 | -0.13245 | 0.01973 |
| Hirundinidae | tmax | -0.47101 | -0.30008 | -0.21305 | 0.305843 | -0.34203 | 0.65741 |
|  | tmin | -0.43538 | -0.38496 | -0.2658 | 0.353808 | 0.130892 | -0.67029 |
|  | rham | 0.442499 | -0.34425 | -0.3689 | -0.23674 | -0.67349 | -0.19991 |
|  | rhpm | 0.393415 | -0.44724 | -0.40856 | -0.01264 | 0.632182 | 0.280106 |
|  | rad | -0.48785 | -0.04403 | -0.21636 | -0.83701 | 0.1123 | 0.008082 |
|  | pre | 0.003222 | -0.6643 | 0.730848 | -0.15652 | -0.0054 | 0.005939 |
| Cardinalidae | tmax | -0.5419 | 0.214733 | -0.13573 | 0.378272 | -0.2815 | 0.647669 |
|  | tmin | -0.56817 | 0.117157 | -0.16751 | 0.356389 | 0.129651 | -0.70113 |
|  | rham | -0.13512 | -0.58759 | -0.36225 | -0.23417 | -0.65585 | -0.14245 |
|  | rhpm | -0.25135 | -0.54962 | -0.29721 | -0.09355 | 0.684868 | 0.261945 |
|  | rad | -0.36108 | 0.446582 | -0.15685 | -0.80214 | 0.046127 | 0.005495 |
|  | pre | -0.41439 | -0.3056 | 0.842235 | -0.15127 | -0.05139 | -0.00288 |
| Setophaga | tmax | -0.49243 | -0.24772 | -0.1951 | -0.33463 | 0.310284 | 0.670696 |
|  | tmin | -0.46642 | -0.32314 | -0.24684 | -0.37064 | -0.06199 | -0.68985 |
|  | rham | 0.404421 | -0.43161 | -0.29396 | 0.233243 | 0.696867 | -0.15401 |
|  | rhpm | 0.330906 | -0.52998 | -0.39399 | -0.00387 | -0.63553 | 0.224685 |
|  | rad | -0.49207 | 0.036526 | -0.31655 | 0.804703 | -0.09359 | 0.004908 |
|  | pre | -0.15742 | -0.60475 | 0.74777 | 0.220644 | -0.04003 | 0.00719 |
| Oenanthe | tmax | -0.47121 | -0.2759 | 0.231385 | 0.297991 | 0.345085 | 0.663642 |
|  | tmin | -0.44017 | -0.36336 | 0.29203 | 0.356935 | -0.1047 | -0.67125 |
|  | rham | 0.444881 | -0.3244 | 0.370876 | -0.24573 | 0.679888 | -0.19149 |
|  | rhpm | 0.398198 | -0.43767 | 0.432607 | 0.013609 | -0.62477 | 0.268704 |
|  | rad | -0.4772 | -0.05476 | 0.214166 | -0.84022 | -0.1317 | 0.009499 |
|  | pre | -0.00359 | -0.70146 | -0.70044 | -0.13136 | 0.004194 | 0.00684 |
| Corvus | tmax | -0.47124 | 0.277373 | 0.231374 | -0.29488 | -0.35093 | 0.661327 |
|  | tmin | -0.43903 | 0.366457 | 0.294893 | -0.3542 | 0.108974 | -0.66982 |
|  | rham | 0.445443 | 0.325013 | 0.364206 | 0.249129 | -0.68025 | -0.19622 |
|  | rhpm | 0.397381 | 0.438793 | 0.434017 | -0.00528 | 0.62112 | 0.274519 |
|  | rad | -0.47838 | 0.058065 | 0.200609 | 0.843219 | 0.128223 | 0.00861 |
|  | pre | 0.000332 | 0.698018 | -0.70586 | 0.120308 | -0.00214 | 0.006938 |
| Vireonidae | tmax | -0.49355 | -0.25321 | -0.183 | 0.35851 | -0.28291 | -0.67099 |
|  | tmin | -0.46564 | -0.33063 | -0.23573 | 0.368739 | 0.096971 | 0.687695 |
|  | rham | 0.402172 | -0.43621 | -0.30369 | -0.17893 | -0.70709 | 0.154143 |
|  | rhpm | 0.334185 | -0.52324 | -0.38986 | -0.0351 | 0.639007 | -0.2302 |
|  | rad | -0.49377 | 0.037445 | -0.3319 | -0.80186 | 0.040252 | -0.00582 |
|  | pre | -0.14952 | -0.60092 | 0.746035 | -0.24347 | 0.025245 | -0.00744 |
| Turdus | tmax | -0.47341 | -0.29885 | 0.191431 | 0.333451 | 0.32159 | -0.65978 |
|  | tmin | -0.4383 | -0.38213 | 0.24162 | 0.363051 | -0.14203 | 0.671941 |
|  | rham | 0.43855 | -0.35756 | 0.375572 | -0.18837 | 0.68247 | 0.193701 |
|  | rhpm | 0.389708 | -0.45319 | 0.403254 | -0.01496 | -0.63589 | -0.27486 |
|  | rad | -0.48945 | -0.04646 | 0.25996 | -0.8273 | -0.07873 | -0.00882 |
|  | pre | -0.00261 | -0.65519 | -0.73057 | -0.19203 | 0.009129 | -0.00593 |

Table S5. Model coefficients, T-values and p-values for linear mixed effects models with seasonal niche overlap as response variable. Migratory status (migratory vs. resident, note resident coefficient values displayed here), breeding location (tropics vs. non-tropics, tropical breeding coefficient values shown here), geographic range size (log-transformed), and their two-way interactions (indicated by *) were included as fixed effects, clade was included as a random effect. Marginal R^2^ describes the proportion of variance explained by the fixed factors. The conditional R^2^ describes the proportion of variance explained by the fixed effects and the random effect (clade). Significant terms are indicated in bold. N= 437 species.

|  | **Seasonal niche overlap** | | |
| --- | --- | --- | --- |
| ***Predictors*** | ***Estimates***  ***(SE)*** | ***T Statistic*** | ***p*** |
| (Intercept) | -1.02 (0.21) | -4.94 | **<0.001** |
| Migratory status (Resident) | 0.54 (0.22) | 2.48 | **0.013** |
| Breeding location (Tropical) | -0.32 (0.20) | -1.60 | 0.109 |
| Log (Range size) | 0.08 (0.01) | 6.31 | **<0.001** |
| Migratory status (Resident) * Breeding location (Tropical) | 0.10 (0.04) | 2.44 | **0.015** |
| Migratory status (Resident) * Log (Range size) | -0.03 (0.01) | -2.11 | **0.035** |
| Breeding location (Tropical) * Log (Range size) | 0.03 (0.01) | 2.02 | **0.044** |
| Random Effects | | | |
| σ^2^ | 0.03 | | |
| τ_00_ _Clade_ | 0.00 | | |
| ICC | 0.03 | | |
| N _Clade_ | 8 | | |
| Observations | 437 | | |
| Marginal R^2^ / Conditional R^2^ | 0.372 / 0.388 | | |

**Table S6.** Results of linear models with seasonal niche overlap as a response variable. Migratory status (migratory vs. resident), breeding location (tropics vs. non-tropics) and clade, as well as their two and three way interactions (indicated by *) were included as fixed effects. Geographic range size (log-transformed) was also controlled for. Significant terms are indicated in bold. N=437.

|  | ***Seasonal niche overlap*** | | |
| --- | --- | --- | --- |
| ***Predictors*** | ***F*** | ***P*** | ***DF*** |
| Migratory status | 4.96 | **<0.05** | 1 |
| Breeding location | 100.73 | **<0.001** | 1 |
| Clade | 5.96 | **<0.001** | 7 |
| Log (Range size) | 133.26 | **<0.001** | 1 |
| Migratory status * Breeding location | 2.73 | 0.098 | 1 |
| Migratory status * Clade | 0.73 | 0.65 | 7 |
| Breeding location * Clade | 1.90 | 0.068 | 7 |
| Migratory status * Breeding location * Clade | 1.15 | 0.33 | 4 |

**Table S7.** Results of linear models testing with seasonal niche overlap as a response variable. Migratory status (migratory vs. resident) and clade, as well as their interaction (indicated by *) were included as fixed effects. Geographic range size (log-transformed) was also controlled for. Significant terms are indicated in bold. N=437).

|  | ***Seasonal niche overlap*** | | |
| --- | --- | --- | --- |
| ***Predictors*** | ***F*** | ***P*** | ***DF*** |
| Migratory status | 4.46 | **<0.05** | 1 |
| Clade | 5.80 | **<0.001** | 7 |
| Log (Range size) | 172.80 | <0.001 | 1 |
| Migratory status * Clade | 0.57 | 0.78 | 1 |

**Table S8.** Summary of phylogenetic generalized least squares (PGLS) analysis with seasonal niche overlap as the response variable. Predictor variables included fixed effects for migratory status (Resident coefficient values shown here), Breeding location (Tropical breeding coefficient values shown here), range size (logged) and the interaction between breeding location and migratory status (indicated by*) whilst considering the phylogenetic effect on these traits. Model summary is mean of analysis carried out on 100 posterior trees selected at random. Mean estimated lambda for the model = 0.087. N = 415

|  | ***Seasonal niche overlap*** | | |
| --- | --- | --- | --- |
| ***Predictors*** | ***Estimate (SE)*** | ***T statistic*** | ***P*** |
| (Intercept) | -0.83 (0.11) | -7.73 | <0.001 |
| Migratory status (Resident) | 0.10 (0.03) | 3.68 | <0.001 |
| Breeding location (Tropical) | 0.08 (0.03) | 2.38 | <0.05 |
| Log (Range size) | 0.07 (0.01) | 1.07 | <0.001 |
| Migratory status (Resident) * Breeding location (Tropical) | 0.07 (0.04) | 1.74 | 0.083 |

**References**

Barrett, G. et al. 2003. The New Atlas of Australian Birds. - CSIRO Publishing.

Borrow, H. and Demey, R. 2001. Birds of Western Africa. - Christopher Helm.

Brazil, M. 2009. Birds of East Asia: China, Taiwan, Korea, Japan and Russia. - Princeton University Press.

Broennimann, O. et al. 2012. Measuring ecological niche overlap from occurrence and spatial environmental data. - Global Ecology and Biogeography 21: 481–497.

del Hoyo, J. et al. 2003- 2011. Handbook of the Birds of the World Alive. Vol 8-16 with updates from www.hbw.com (2016-2017). - Lynx Edicions.

Dickinson, E. C. et al. 2010. A guide to the birds of the Philippines. - Oxford University Press.

Eyres, A. et al. 2017. Quantification of climatic niches in birds: Adding the temporal dimension. - Journal of Avian Biology 48: 517–1531.

Fry, C. H. and Urban, E. K. 1992-2004. The Birds of Africa. Vol. IV-VII. - Academic Press/ Christopher Helm.

Holt, B. G. et al. 2013. An update of Wallace’s zoogeographic regions of the world. - Science 339: 74–77.

Myers, S. 2010. Borneo, A Field Guide to the Birds of. - New Holland Publishers.

Orme, C. D. L. et al. 2005. Global hotspots of species richness are not congruent with endemism or threat. - Nature 436: 1016–1019.

Rasmussen, P. C. and Anderton, J. C. 2012. Birds of South Asia. The Ripley Guide. - Michigan State University and Lynx Edicions.

Ridgely, R. S. et al. 2012. Digital Distribution Maps of the Birds of the Western Hemisphere, version 3.0. - NatureServe in press.

Svensson, L. et al. 2010. Collins Bird Guide: The most complete Guide to the Birds of Britain and Europe. - Harper Collins.

Warren, D. L. et al. 2008. Environmental Niche Equivalency Versus Conservatism: Quantitative Approaches to Niche Evolution. - Evolution 62: 2868–2883.
